# Genomic Analyses of *Bifidobacterium moukalabense* Reveal Adaptations to Frugivore/Folivore Feeding Behavior

**DOI:** 10.3390/microorganisms7040099

**Published:** 2019-04-04

**Authors:** Takahiro Segawa, Satoshi Fukuchi, Dylan Bodington, Sayaka Tsuchida, Pierre Philippe Mbehang Nguema, Hiroshi Mori, Kazunari Ushida

**Affiliations:** 1 Center for Life Science Research, University of Yamanashi, 1110 Shimokato, Chuo, Yamanashi 409-3898, Japan; tsegawa@yamanashi.ac.jp; 2 Faculty of Engineering, Maebashi Institute of Technology, Maebashi, Gunma 371-0816, Japan; sfukuchi@maebashi-it.ac.jp; 3 Department of Biological Sciences, Tokyo Institute of Technology, Tokyo 152-8550, Japan; bodington@gmail.com; 4 Chubu University Academy of Emerging Sciences, Kasugai, Aichi 487-8501, Japan; s_tsuchida@isc.chubu.ac.jp; 5 Graduate School of Life and Environmental Sciences, Kyoto Prefectural University, Kyoto 606-8522, Japan; 6 Research Institute of Tropical Ecology, Libreville BP 13354, Gabon; mbehangnguema@yahoo.fr; 7 Center for Information Biology, National Institute of Genetics, 1111 Yata, Mishima, Shizuoka 411-8540, Japan; hmori@nig.ac.jp

**Keywords:** *Bifidobacterium moukalabense*, genomic characteristics, wild gorillas, wild chimpanzees, wild forest elephants

## Abstract

Despite the essential role of *Bifidobacterium* in health-promoting gut bacteria in humans, little is known about their functions in wild animals, especially non-human primates. It is difficult to determine in vivo the function of Bifidobacterium in wild animals due to the limited accessibility of studying target animals in natural conditions. However, the genomic characteristics of *Bifidobacterium* obtained from the feces of wild animals can provide insight into their functionality in the gut. Here, we analyzed the whole genomes of 12 *B. moukalabense* strains isolated from seven feces samples of wild western lowland gorillas (*Gorilla gorilla gorilla*), three samples of wild central chimpanzees (*Pan troglodytes troglodytes*) and two samples of wild forest elephants (*Loxodonta cyclotis*) in Moukalaba-Doudou National Park, Gabon. In addition, we analyzed the fecal bacterial communities of six wild western lowland gorillas by meta 16S rRNA gene analyses with next generation sequencing. Although the abundance of the genus *Bifidobacterium* was as low as 0.2% in the total reads, a whole genome analysis of *B. moukalabense* suggested its contribution digestion of food and nutrition of frugivore/folivore animals. Specifically, the whole genome analysis indicated the involvement of *B. moukalabense* in hemicellulose degradation for short chain fatty acid production and nucleic acid utilization as nitrogen resources. In comparison with human-associated *Bifidobacterium* spp., genes for carbohydrate transport and metabolism are not conserved in these wild species. In particular the glycosidases, which are found in all 12 strains of *B. moukalabense*, were variably detected, or not detected, in human-associated species.

## 1. Introduction

The gut microbiome is composed of an immense diversity of microorganisms [1], and recent studies have shown their essential roles in host health [2,3,4,5,6,7]. Among these, the genus *Bifidobacterium* is the unique Actinobacteria, which is adapted to animal intestinal tracts. Bifidobacteria have been commonly recognized as health-promoting gut bacteria in many health-oriented research studies [8,9,10,11,12]. At present, the genus *Bifidobacterium* comprises 70 established species with 10 subspecies according to LPSN (http://www.bacterio.net/bifidobacterium.html). Within these 70 species, human-associated species of *Bifidobacterium* are abundant. In contrast, other animal species often carry bifidobacteria with much lower levels of diversity [13]. In fact, the human gut normally harbors *Bifidobacterium* as one of the most abundant bacterial genera (~10%) [14], and to date, 12 species belonging to *B. adolescentis*-group, *B. longum*-group or *B. scardovi*-group and *B. bifidum* have been recognized as human-associated bifidobacteria [15]. The wide phylogenetic variety of human-associated Bifidobacterium spp. suggests their adaptation to the wide range of eating habits of humans [16].

Several comparative genomic studies of *Bifidobacterium* have been conducted to assess the genomic characteristics of this particular genus in terms of adaptation to the human intestinal tract [17,18,19]. The discussion mostly focused on carbohydrate metabolism and adaptation to a wide variety of dietary carbohydrates. In addition, bile acid tolerance of *Bifidobacterium* spp. has been identified in some of the probiotic strains [20,21,22]. The wide variety of food carbohydrates and wide phylogenetic diversity of human-associated *Bifidobacterium* spp. may be related.

*B. moukalabense* was first isolated from a wild western lowland gorilla (*Gorilla gorilla gorilla*) [23]. According to the 16S rRNA phylogeny, *B. moukalabense* belongs to the *B. adolescentis*-group and is closely related to *B. catenulatum* and *B. pseudocatenulatum*. The draft genome sequence reported later on this strain GG01^T^ indicated that this strain is close to *B. dentium*, the other member of the *B. adolescentis* group [24]. Genetic comparisons between *B. moukalabense* and those from human associated *Bifidobacterium* can address the question of why this species has not been identified from human microbiota, while it was identified in Homininae primates.

Since our first isolation of this particular *B. moukalabense* [23], we have successfully isolated several other strains of *B. moukalabense* from wild western lowland gorillas, wild central chimpanzees (*Pan troglodytes troglodytes*) and wild forest elephants (*Loxodonta cyclotis*) in the same forest in Moukalaba-Doudou National Park, Gabon. In this study, we carried out genomic analyses of 12 strains involving GG01^T^ to reveal the genomic characteristics by comparing the annotated gene functionality with those of human-associated *Bifidobacterium* species. The genomic differences and similarities between *B. moukalabense* and human-associated *Bifidobacterium* spp. suggested functional adaptations of *B. moukalabense* to the frugivore/folivore feeding behavior of the host.

## 2. Materials and Methods

### 2.1. Isolation and Identification of Bifidobacteria

*Bifidobacteria* were isolated from the feces of wild western lowland gorillas, central chimpanzees and forest elephants in Moukalaba-Doudou National Park in Gabon (Table 1). Seven gorilla feces samples and three chimpanzee feces samples were obtained by closely (10 to 15 m) following a troop of gorilla or a troop of chimpanzee. Therefore, it was confirmed by visual observation that all feces were defecated by different individuals. Two elephant feces samples were obtained from a troop of three or four individuals. Since the wild forest elephants in this forest are not accustomed to human observers, it was difficult to follow them closely. Accordingly, it was not possible to conclude that these two elephant feces samples were defecated by different individuals, although the two feces samples were found within about 20 m distance from each other. The bifidobacterial strains used in this study were isolated and identified as described previously [23]. Briefly, loopful feces was applied to a Bifidobacterium-selective (BS) agar medium supplemented with 5% defibrinated horse blood at the sampling site. The plates were then quickly placed under anaerobic conditions in a pouch with an AnaeroPack (Mitsubishi Gas Chemical, Tokyo, Japan). After returning to the camp site, the pouches were placed in a styrene foam box with hand warmers, which heat the plates. The temperature was controlled manually in order to maintain storage, as far as this was possible, at 37 °C. After returning to the laboratory in Libreville, Gabon, the developed colonies were purified and the colony purity was confirmed microscopically. Seven, three and two isolates were respectively obtained from seven freshly defecated gorilla feces samples, three freshly defecated chimpanzee feces samples and two freshly defecated elephant feces samples. Isolates were grown for 24h on the same plate medium, and developed colonies were subjected to DNA isolation using the previously described methods, with bead beating in a 5% (*v*/*v*) Triton X-100 [23].

PCR amplification of the 16S rRNA gene was performed using EX-*Taq* polymerase (Takara, Kyoto, Japan) and primers 27F and 1492R [25] with a thermal cycler (iCycler, BioRad, Tokyo, Japan). The PCR conditions were as follows: 3 min of initial denaturation at 95 °C, 30 cycles at 95 °C for 30 s, 50 °C for 30 s and 72 °C for 90 s, and a final extension at 72 °C for 10 min. The PCR products were purified using Wizard SV Gel and PCR Clean-Up System (Promega, Madison, WI, USA). Sequencing was achieved using Big Dye Terminator v3.1 and an ABI 3130xl automatic sequence analyzer (Applied Biosystems, Foster City, CA, USA). BLASTN searches were performed to identify the closest sequences in the NCBI-nt database.

### 2.2. Genome Library Construction and Illumina Sequencing

An aliquot of DNA (40–80 ng) from each isolate was sheared to a target peak size of 550 bp using the Covaris S220 Focused-Ultrasonicator system (Covaris, Woburn, MA, USA) according to the manufacturer’s recommendations. To generate DNA sequencing libraries for high-throughput DNA sequencing, a TruSeq DNA PCR-Free Library Preparation Kit (Illumina, San Diego, CA, USA) was used according to the manufacturer’s instructions. The library products were isolated in agars gels (size of 500–800 bp) and purified by use of NucleoSpin Gel and PCR Clean-up (Takara, Japan). The sequencing library was used as a template for paired-end sequencing using a MiSeq Reagent Kit v3 (600 cyc) and the MiSeq desktop sequencer (Illumina, San Diego, CA, USA). Read files (fastq.gz) sequence reads were generated (average 927,054 read pairs per strain) using MiSeq Reporter software version 2.3.32 (Illumina, San Diego, CA, USA).

### 2.3. Phylogenetic Tree Construction Based on Core-Genome

We discarded the Illumina MiSeq platform reads that contained ambiguous nucleotides, low qualily fastq reads (< average QV 25) and those mapped to the PhiX genome sequence. We also removed Illumina adapter sequences. These filtration steps were performed using Prinseq version 0.20.4 [26], Bowtie 2 version 2.1.0 [27] and Trimmomatic version 0.32 [28] with default parameters, respectively. Filtered sequencing reads were assembled into contigs using IDBA [29], a de novo assembler, with default parameters. Gene prediction and annotation was performed using Prokka [30], a tool for rapid annotation of prokaryotic genomes. The predicted genes from all sequenced strains and all reference strains were clustered into homologous gene families using get_homologues [31], which uses all-against-all BLASTP to define putative pairs of orthologs or recent paralogs. We compared the genomes of the *B. moukalabense* group from all isolates with human-associated *Bifidobacterium*, i.e., *B. catenulatum*, *B. dentium*, *B. pseudocatenulatum*, and *B. longum*, to reveal potentially adaptive function of *Bifidobacterium* in non-human frugivore/folivore animals. *B. longum*, which constitutes a neighboring phylogenetic group of the *B. adolescentis* group [15] was involved in this genomic analysis as a form of out-group. Clustering of genes was performed using orthoMCL [32] with a max E-value of 10^−5^ and a minimum coverage of 75% in the BLASTP alignments. The core genome was constructed using clusters of only single-copy genes from all sequenced and reference strains, and a pangenome matrix was constructed to manually inspect the non-core homologues. *B. catenulatum* was used as the reference for annotation for all homologous clusters in the core genome set, while clusters outside the core genome (*B. dentium*, *B. pseudocatenulatum* and *B. longum*) were manually curated by inspecting the annotations for all strains present in the cluster. The proteins were classified by the COG functional category [33]. The genomes studied are listed in Table S1. The core-genome DNA sequences were aligned using the program MAFFT and phylogenetic relationships among *B. moukalabense*, *B. denitium*, *B. catenulatum* and *B. pseudocatenulatum* were reconstructed based on their genomic data with the maximum likelihood method and the Bayesian method. The nucleotide substitution models were evaluated by the criterion of AIC, AICc, and BIC with the ModelFinder, and the GTR+I+Γ model was selected as the best model in all criterion. Maximum likelihood tree was reconstructed using RAxML version 8.2.10 [34] with the GTR+I+Γ model. To evaluate the confidences of the internal branches, the bootstrap method was applied by the rapid bootstrap algorithm [34] with 1000 replications. The Bayesian tree was reconstructed using the MrBayes version 3.2.6 with the GTR+I+Γ model under the two independent sets of the four simultaneous chains of the MCMC with the length of 100,000 generations. The trees and parameters were sampled every 100 generations. The first 10,000 generations were discarded as the burn-in. Carbohydrate-active enzymes were identified based on similarity to the carbohydrate-active enzyme (CAZy) database [35] using HMMER 3.1 software [36] with a max E-value cutoff of 1 × 10^−18^.

### 2.4. Orthologous Abundance Comparison of the Human Metagenome-Derived Bifidobacterium Genomes

The 3973 draft *Bifidobacterium* genomes, derived from 9428 human metagenome samples, were obtained from a database (http://opendata.lifebit.ai/table/SGB) [37]. Protein coding genes (6,105,571 genes) from the 3973 draft *Bifidobacterium* genomes were predicted using Prodigal version 2.6.3 with -p meta parameter [38]. The 5832 representative amino acid sequences of each orthologous cluster in the presently determined 12 genome data of *B. moukalabense* were searched for against the 6,105,571 amino acid sequences using MMseqs2 with -s 4 parameter [39], and filtered out with identity <40% hits. The number of genomes which has homologous genes of each orthologous cluster was calculated.

### 2.5. High Throughput Sequencing of 16S rRNA Genes on Feces Samples

Feces samples from gorillas were collected in August 2011 in Moukaraba-Doudou National Park, as indicated elsewhere [23]. Briefly, freshly defecated feces were collected from six adult gorillas and transferred in DNA conservation solution [40]. Partial 16S rRNA gene sequences including V3 and V4 regions were amplified using primers Bakt_341F and Bakt_805R [41] with Illumina overhang adaptor sequences attached to their 5′ ends. 16S rRNA gene amplicon libraries were constructed as described previously [42]. The amplicons with Illumina overhang adaptor sequences were triplicated and each amplicon was polled before index PCR using a Nextera XT Index kit (Illumina, San Diego, CA, USA). The resulting DNA was used as a template for paired-end sequencing using a MiSeq Reagent Kit v3 (600 cycles) and the MiSeq desktop sequencer. Genus compositions were inferred using VITCOMIC2 [43].

### 2.6. Ethics

The feces of wild gorillas, chimpanzees, and elephants were collected non-invasively, as indicated elsewhere [23]. The sampling was conducted with the collaboration of local veterinary staff within the SATREPS program (J. Yamagiwa, 2009–2014) under R/D DOCUMENTS between the Japanese International Cooperation Agency (JICA) and the Gabonese Government (Ministère de l’Enseignement Supérieur de la Recherche Scientifique et du Développement Technologique, Centre National de la Recherche Scientifique et Technologique and L’Institut de Recherche en Écologie Tropicale) and MOU between Kyoto University and L’Institut de Recherche en Écologie Tropicale. Permission of access to these wild animals and of DNA use was obtained from the Centre National de la Recherche Scientifique et Technologique as No. 0610/MENESRSI/CENAREST/CG/CST/CRI (26/10/2009), No.0743/MENESRSI/CENAREST/CG/CST/CRI (20/10/2010), No. 306/MENESRSI/CENAREST/CG/CST/CRI (14/10/2011).

## 3. Results

The draft genome sequences of the 12 strains included 18 to 33 contigs with varying size up to 2.40–2.59 Mbps (Table S1) with relatively good N50 values (0.14 to 1.56 Mbp). Estimated genome sizes of 11 strains of *B. moukalabense* were 2.5 Mb ± 0.59 (mean ± SD) with nearly 59.9% GC content. These genomes harbor from 1919 to 2143 coding sequences (CDSs), from 57 to 64 rRNAs, and 4 or 5 tRNAs. No plasmid associated genes were detected.

**Figure 1:**
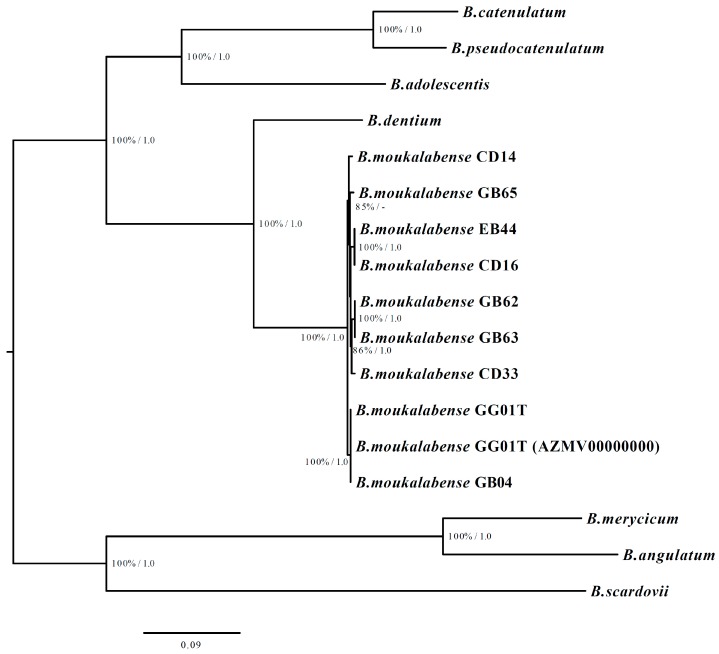
The maximum likelihood tree of *B. moukalabense* and its related species. The nodal numbers represent the bootstrap values (*left*) and Bayesian posterior probability (*right*). The branch lengths are proportional to the numbers of the nucleotide substitutions.

*B. moukalabense* was confirmed to be phylogenetically close to *B. catenulatum* and *B. pseudocatenulatum* and is a monophyletic species belonging to the *B. adolescentis*-group by 16S rRNA gene analysis (Figure S1). However, the pangenome phylogenetic tree shows that *B. moukalabense* is close to *B. dentium* rather than *B. catenulatum* and *B. pseudocatenulatum* (Figure 1).

**Figure 2:**
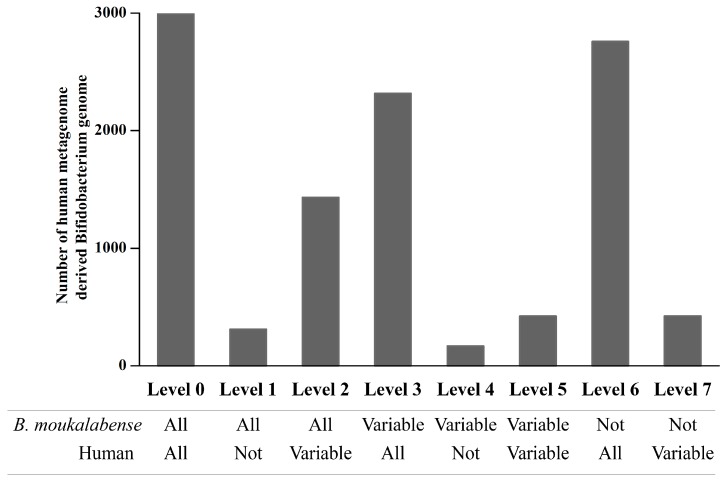
Numbers of shared orthologs derived from genomes of *Bifidobacterium moukalabense* and meta-bifidobacterial genomes reconstructed from human metagenome data. The 3973 draft *Bifidobacterium* genomes were derived from 9428 human metagenome samples (37). Protein coding genes (6,105,571 genes) from the 3973 draft *Bifidobacterium* genomes were predicted. For details, see text.

The total number of proteins in this study was 39,774. They were organized into 5832 clusters, hereafter called “orthologous clusters”. We categorized these proteins and orthologous clusters into seven levels according to the conservation level when compared with human-associated *Bifidobacterium such as B. catenulatum, B. pseudocatenulatum, B. dentium,* and *B. longum* (Tables S2 and S3). The frequencies of protein and orthologous clusters are shown in Figure S2. The remarkable feature in this analysis is that quite small numbers of proteins have been acquired in the human-associated species after divergence of these species and *B. moukalabense* (the numbers of proteins and orthologous clusters are 35 and 5 in level 6, see Figure S2). On the other hand, a considerable number of genes have been lost in the human-associated species as shown in level 1 and 2. In the comparison between 12 genomes of *B. moukalabense* (PRJDB7909) and 3973 meta-bifidobacterial genomes reconstructed from a human metagenome database [37], the conservation of genes was categorized into the 7 levels. The conservation pattern is shown in Figure 2, and is quite similar to those obtained by the comparison of 13 *B. moukalabense* genomes (PRJDB7909 and AZMV00000000) and seven human-associated *Bifidobacterium*.

**Figure 3:**
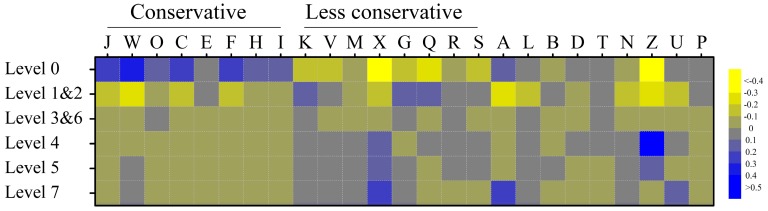
The frequency of the orthologs at different conservation level broken down by COG category.

The conservation level, broken down by the COG category, is shown in Figure 3 and Table S4. The conservative functional categories are involved in essential biological activity, namely translation (J), energy production (C), amino acid, nucleotide, coenzyme, and lipid transport (E, F, H, and I respectively). On the other hand, the less conservative categories contain genes varying between *B. moukalabense* and human-associated species: defense mechanisms (V), mobilome (X), and secondary metabolites metabolism (Q). The category of transcription (K), cell wall and membrane (M) and carbohydrate transport and metabolism (G) are also the less conservative.

When the genes were categorized by conservation levels between human-associated species and *B. moukalabense*, those in conservation levels 1 and 2 that are present in all *B. moukalabense* strains, and were variably present or not present in the human-associated species, were particularly over-represented in this functional category, carbohydrate transport and metabolism, showing that the genes involved in the carbohydrate metabolism are heterogeneous in the human-associated species (Tables S5–S8). Upon investigation of the gene content in category G, a remarkable feature is that the proportion of glycosidases in levels 1 and 2 was larger than the proportion of all of the other proteins in category G (Figure S3). The annotation of the orthologous cluster in category G and conservation level 2 is shown in Table S5. These glycosidases are mostly involved in the degradation of oligosaccharides to obtain energy from sugars.

Although the proteins associated with amino acid metabolism (E) and nucleotide metabolism (F) are conservative (Figure 3), a clear gene loss in the human-associated species is observed. We listed genes which are lost in human-associated species but which are present in all of the *B. moukalabense* strains (level 1 genes) in Table S6. In this table, a series of genes in a pathway associated with allantoin/hidantoin (orthologous cluster ID from 1792 to 1798) are lost in all human-associated species. As shown in Figure S4, these proteins are mapped on the pathway from allantoin to glycine in the KEGG pathway database [44].

Bacterial communities were also determined by meta 16S rRNA gene analysis with a Miseq platform for six feces samples collected from six wild western lowland gorillas. The abundance of the genus *Bifidobacterium* in fecal microbiota was calculated to be as low as 0.24% ± 0.15 (mean ± SD) in wild western lowland gorillas (Table S9).

## 4. Discussion

In contrast to humans, *Bifidobacterium* is not recognized as the most prevalent bacterial group in non-human primates, particularly in wild individuals. In fact, only *B. angulatum*-like strains have been isolated from wild western chimpanzees (*Pan troglodytes verus*) in Bossou, Guinea and *B. moukalabense* is the sole *Bifidobacterium* isolated from wild western lowland gorillas [23,45]. This latter species was sole *Bifidobacterium* isolated from wild central chimpanzees in this study. However, only low levels (0.24% abundance) of the genus *Bifidobacterium* were recorded for six gorilla feces samples taken from the Moukalaba-Doudou National Park. Like wild western lowland gorillas, wild central chimpanzees showed low levels of *Bifidobacterium* in their gut microbiota [46]. It is noteworthy that *B. moukalabense* was also isolated from wild forest elephants in the same forest, although low levels of *Bifidobacterium* in their gut were reported [46,47]. Since forest elephants show similar feeding behavior to gorillas, and to a lesser extent, to chimpanzees [48,49,50], the sharing of the same species of *Bifidobacterium* by western lowland gorillas, central chimpanzees and forest elephants suggests that *B. moukalabense* is associated with frugivore/folivore feeding.

*B. moukalabense* belong to the *B. adolescentis* group which comprises *B. dentium*, *B. catenulatum* and *B. pseudocatenulatum* associated with the human gut. Captive-bred gorillas and chimpanzees harbored *B. dentium*, *B. catenulatum* and *B. pseudocatenulatum* instead of *B. angulatum* or *B. moukalabense* [51,52]. Any single species of the non-human Homininae in the natural condition is unlikely to host *Bifidobacterium* with such high density and divergency as in humans, as suggested by the present study (Table S9) and by previous studies [45,46,47,51]. An important point of our findings seems to be the fundamental differences in gene varieties, particularly in fiber degradation and nucleic acid metabolism between *B. moukalabense* and its phylogenetic neighbors, *B. dentium*, *B. catenulatum* and *B. pseudocatenulatum*. *B. moukalabense* is distinguished from *B. dentium*, *B. catenulatum* and *B. pseudocatenulatum* in the *B. adolescentis* group by the presence of a considerable number of unique genes. These unique genes may be important for the functional traits of *B. moukalabense* in the intestine of frugivore/folivores. The genome of *B. moukalabense* uniquely carries a range of carbohydrases (Tables S4 and S10, Figure S5); these carbohydrases are apparently homologs of those found in other intestinal bacteria such as *Bacteroides thetaiotaomicron*. Many of these genes are related to fiber degradation, namely hemicellulose degradation. Such a biased distribution of genes for hemicellulose degrading enzymes indicates that *B. moukalabense* may be related to the function of the frugivore/folivore food habit of its host.

Other strongly biased distributions of genes concerning nitrogen-scavenging capacity, such as nucleic acid catabolism, may also reflect the frugivore/folivore food habit of the host, in which the limitation of protein, i.e., amino acid, availability in the gut is expected (Table S1). Similar trends were observed in *B. thermacidophilum* genomes; domestic pig isolates apparently lost the genes for endoglucanase, beta-glucosidase, chitinase, an endoxylanase-related protein, beta-mannosidase, and pullulanase in comparison with wild boar isolates [53]. Instead, starch-related digestive enzymes such as maltodextrin glucosidase and alpha-glucosidase were detected only in pig isolates [53]. Wild boar isolates, thus, are apparently associated with fiber-rich omnivorous feeding behavior. In fact, cellulose and xylan comprise the major part of plant carbohydrates of the above-ground part, which comprises 70% of the plant materials consumed by wild boars [54]. Similarly, the unique presence of genes for fiber degradation may explain the association of *B. moukalabense* with a predicted fiber-rich intestinal environment found in wild boar isolates of *B. thermacidophilum*. The unique presence of genes for nucleic acid utilization in *B. moukalabense* explains its nitrogen scavenging ability under predicted protein- and amino acid-limited conditions in the intestinal environment. Our results are consistent with the nature of the diets of gorillas and elephants, and to a lesser extent, with that of chimpanzees, which are composed primarily of carbohydrates and are generally limited in protein [55]. The aforementioned differences in genomes between *B. moukalabense* with 7 known genomes of human-associated *Bifidobacterium* spp. were confirmed by a comparison between *B. moukalabense* and reconstructed meta bifidobacterial genomes. Since the meta-bifidobacterial genomes were reconstructed from huge data sets (9428 human metagenome samples), they can represent the human gut microbiome under a wide variety of feeding conditions.

In conclusion, *B. moukalabense* is a unique *Bifidobacterium* which is specialized to frugivore/folivore feeding habits of its host. In this context, this *Bifidobacterium* can reside in the gut of the typical herbivore animal like African forest elephants. But adaptation to frugivore/folivore feeding habits may not have been advantageous in omnivore human ancestors, who developed carnivore food habits [56].

## Figures and Tables

**Table 1:** *Bifidobacterium* strains in this study.

Bacterial Taxon	Strain Name	Source of Isolate	Common Name	Location of Collection	Date of Isolation	Collected by	Acession No.
*Bifidobacterium moukalabense*	GG01T (JCM18751, DSM27321)	*Gorilla gorilla gorilla*	Western Lowland Gorilla	2°19′38″S 10°34′02″E	24/11/2010	K. Ushida and P.P.Nuguma	PRJDB7909, AZMV00000000
	GB01	*Gorilla gorilla gorilla*	Western Lowland Gorilla	2°19′53″S 10°34′06″E	28/10/2009	K. Ushida and P.P.Nuguma	PRJDB7909
	GB03	*Gorilla gorilla gorilla*	Western Lowland Gorilla	2°19′51″S 10°34′19″E	01/11/2009	K. Ushida and P.P.Nuguma	PRJDB7909
	GB04	*Gorilla gorilla gorilla*	Western Lowland Gorilla	2°19′53″S 10°34′21″E	24/11/2010	K. Ushida and P.P.Nuguma	PRJDB7909
	GB62	*Gorilla gorilla gorilla*	Western Lowland Gorilla	2°20′02″S 10°34′05″E	25/11/2010	K. Ushida and P.P.Nuguma	PRJDB7909
	GB63	*Gorilla gorilla gorilla*	Western Lowland Gorilla	2°19′49″S 10°34′10″E	26/11/2010	K. Ushida and P.P.Nuguma	PRJDB7909
	GB65	*Gorilla gorilla gorilla*	Western Lowland Gorilla	2°19′50″S 10°34′05″E	27/11/2010	K. Ushida and P.P.Nuguma	PRJDB7909
	CD14	*Pan troglodytes troglodytes*	Central chimpanzee	2°23′08″S 10°33′14″E	29/11/2011	S. Tsuchida and P.P Nguema	PRJDB7909
	CD16	*Pan troglodytes troglodytes*	Central chimpanzee	2°23′08″S 10°33′14″E	29/11/2011	S. Tsuchida and P.P Nguema	PRJDB7909
	CD33	*Pan troglodytes troglodytes*	Central chimpanzee	2°23′08″S 10°33′14″E	29/11/2011	S. Tsuchida and P.P Nguema	PRJDB7909
	EB43	*Loxodonta cyclotis*	African forest elephant	2°20′12″S 10°34′31″E	01/11/2009	K. Ushida and P.P.Nuguma	PRJDB7909
	EB44	*Loxodonta cyclotis*	African forest elephant	2°20′12″S 10°34′31″E	01/11/2009	K. Ushida and P.P.Nuguma	PRJDB7909
*Bifidobacterium catenulatum*	DSM 16992	*Homo sapiens*	Human				ID_BIFCAT_00411
*Bifidobacterium dentium*	ATCC 27678	*Homo sapiens*	Human				ID_BIFDEN_01570
*Bifidobacterium dentium*	ATCC 27679	*Homo sapiens*	Human				ID_HMPREF0168_0306
*Bifidobacterium dentium*	Bd1	*Homo sapiens*	Human				ID_BDP_1602
*Bifidobacterium dentium*	JCVIHMP022	*Homo sapiens*	Human				ID_HMPREF9003_0584
*Bifidobacterium longum*	DJO10A	*Homo sapiens*	Human				ID_BLD_0124
*Bifidobacterium pseudocatenulatum*	DSM 20438	*Homo sapiens*	Human				ID_BIFPSEUDO_03077

## Data Accessibility

The high-throughput sequencing data have been submitted to the DDBJ Sequence Read Archive under accession number PRJDB7909 and PRJDB7910. The *B. moukalabense* orthologous clusters in this study are available from http://gorilla19.paleogenome.jp.

## Supplementary Materials

The following are available online at https://www.mdpi.com/2076-2607/7/4/99/s1, Figure S1: Neighbor joining phylogenetic tree of *Bifidobacterium* spp. based on 16S rRNA gene sequence; Figure S2: Frequency of the conservation levels of proteins and orthologous clusters; Figure S3: Frequency of the carbohydrate metabolism and transport (category G) proteins; Figure S4: Allantoin metabolism pathway in the KEGG map; Figure S5: Bray-Curtis dissimilarity based on the Carbohydrate Active Enzymes (CAZy) system; Table S1: Genome statistics of *Bifidobacterium moukalabense* in this study; Table S2: Conservation level of proteins and orthologous clusters into the 7 levels in this study; Table S3: COG category list; Table S4: The number of ortholog clusters and proteins of the conservation levels broken down into the COG functional categories; Table S5: The list of the level 2 ortholog clusters within COG category G (carbohydrate metabolism and transport); Table S6: The list of level 1 ortholog clusters with the COG annotation; Table S7: The list of level 2 ortholog clusters with the COG annotation; Table S8: The list of level 6 ortholog clusters with the COG annotation; Table S9: Ratio (%) of fecal microbial community by 16S rRNA gene analysis; Table S10: Classification according to the Carbohydrate Active Enzymes (CAZy) system.
