# Supplementary material for: Genomic Analyses of Bifidobacterium moukalabense Reveal Adaptations to Frugivore/Folivore Feeding Behavior
Source: Microorganisms. 2019 Apr 4;7(4):99. doi: 10.3390/microorganisms7040099 (PMC6518056; doi:10.3390/microorganisms7040099)
Supplement: Supplementary file 1 [file microorganisms-07-00099-s001.pdf]

Supplementary Information

Genomic analyses of *B*ifidobacterium moukalabense reveal adaptations to fruigivore/folivore feeding behavior

Takahiro Segawa, Satoshi Fukuchi, Dylan Bodington, Sayaka Tsutida, Pierre Philippe Mbehang Nguema, Hishiro Mori, Kazunari Ushida

This PDF file includes:

Supplementary Figures 1-5

Supplementary Tables 1-10

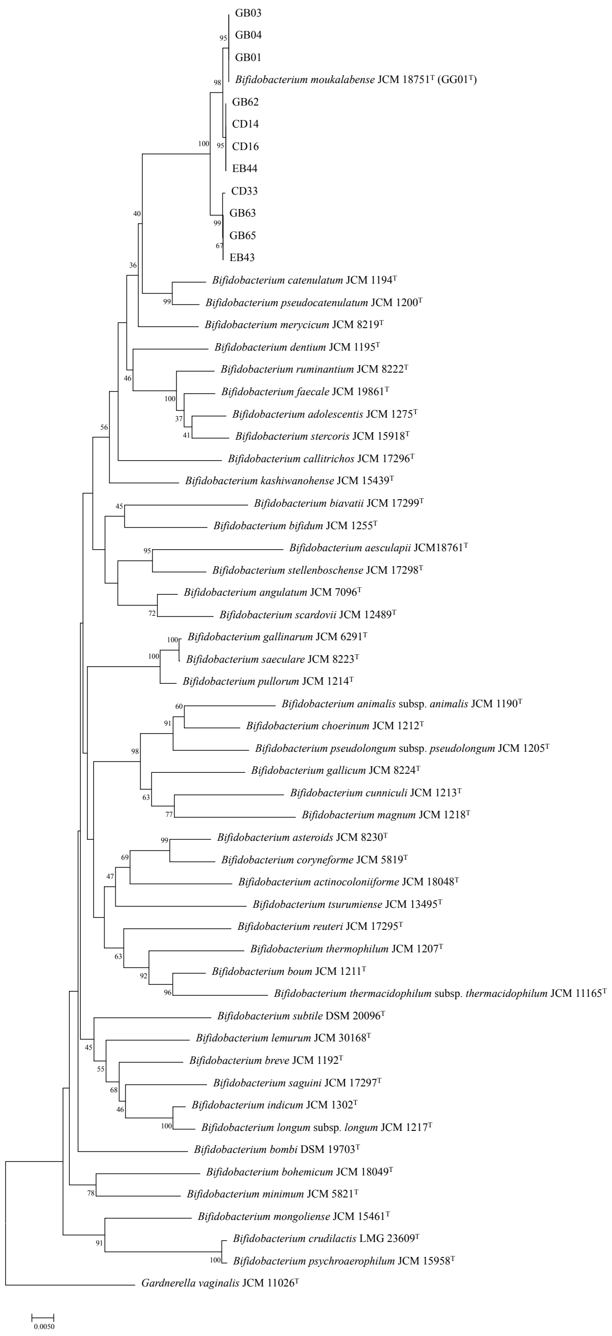

Supplementary Figure 1: **Neighbor joining phylogenetic tree of *B*ifidobacterium spp. based on 16S rRNA gene sequence.** Tree was constructed with 1000 bootstrap replication using MEGA version 5. The sequences were retrieved from DDBJ/GenBank/EMBL. Bar represents 0.02 substitution per nucleotide position.

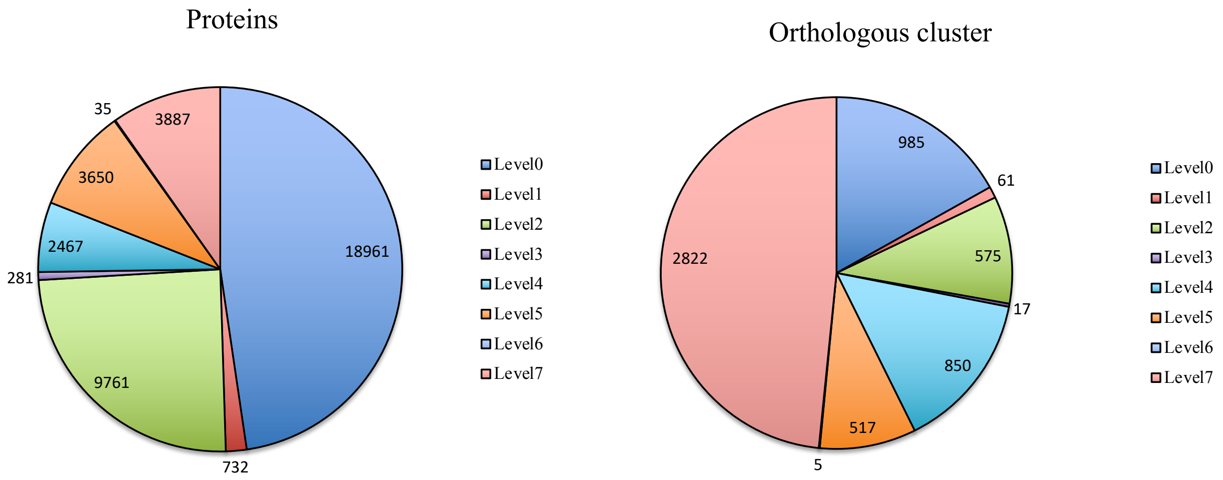

Supplementary Figure 2: **Frequency of the conservation levels of proteins and orthologous clusters.**

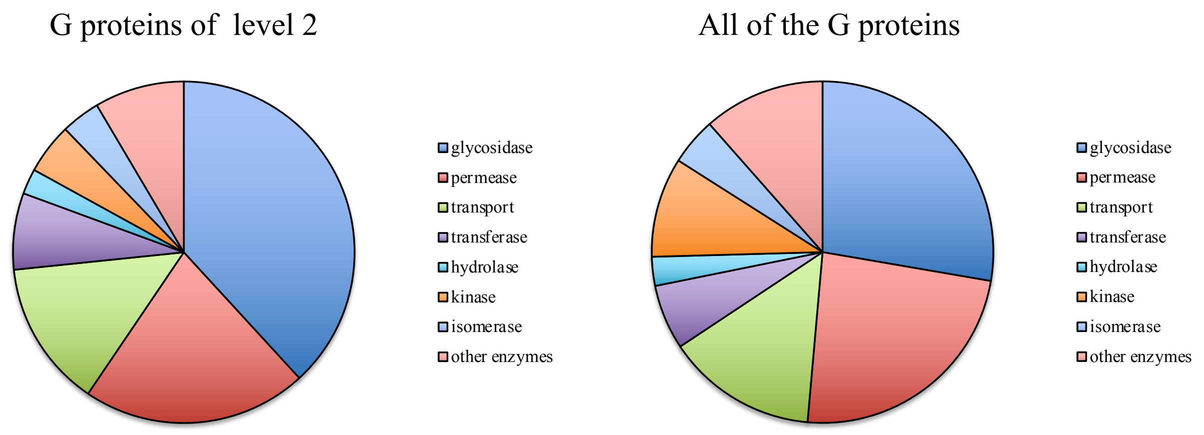

Supplementary Figure 3: Frequency of the carbohydrate metabolism and transport (category G) proteins.

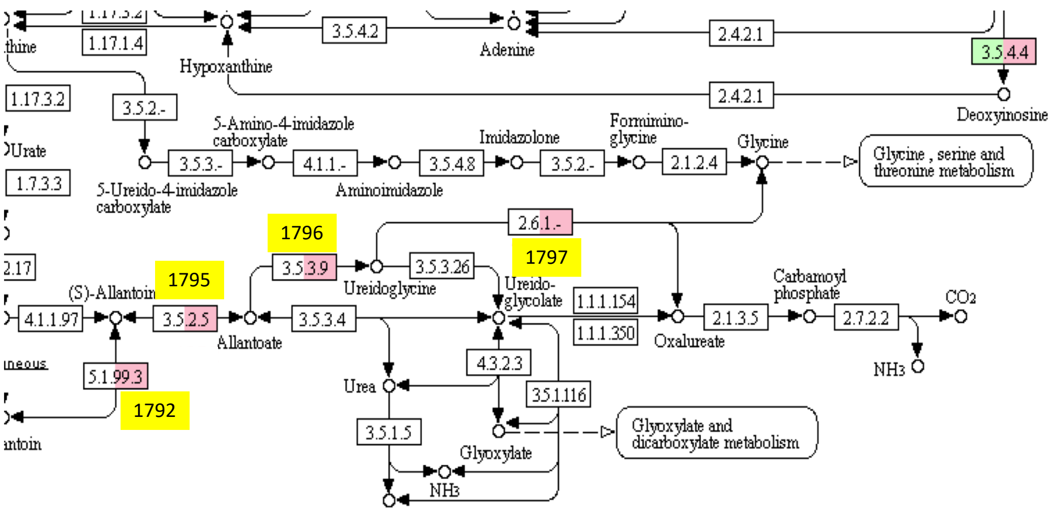

Supplementary Figure 4: **Allantoin metabolism pathway in the KEGG map.**

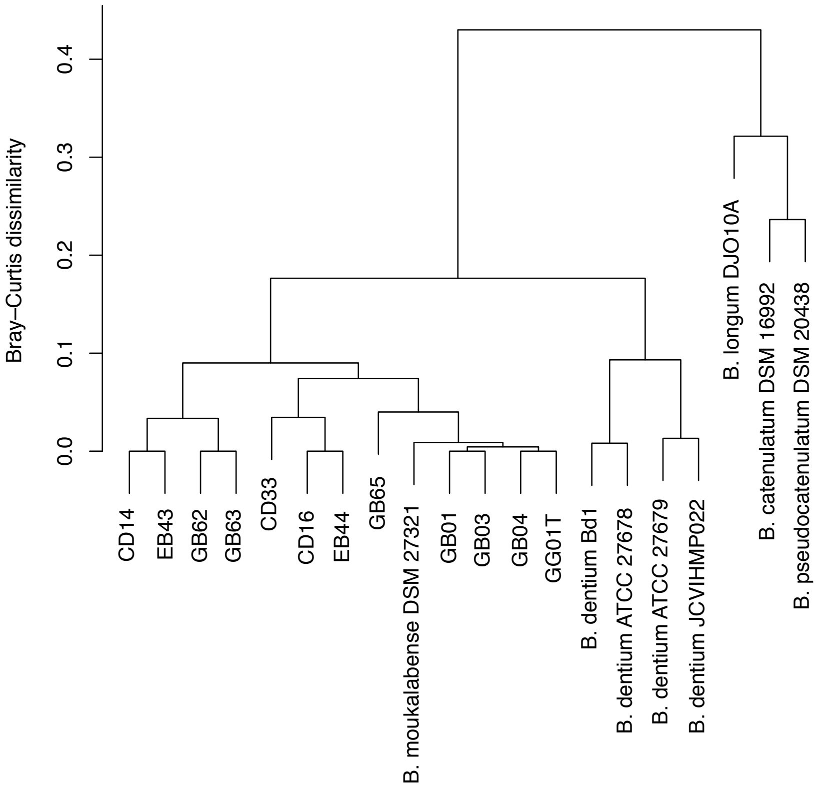

Supplementary Figure 5: **Bray-Curtis dissimilarity based on the Carbohydrate Active Enzymes (CAZy) system.**

Supplementary Table 1: Genome statistics of *Bifidobacterium moukalabense* in this study.

	CD14	CD16	GB01	CD33	GB03	EB43	EB44	GB04	GB62	GB63	GB65	GG01T
Scaffold (>= 1000 bp)	18	23	23	33	20	18	27	22	24	30	31	22
Total length (Scaffold >= 1000 bp)	2,451,300	2,405,620	2,523,078	2,515,748	2,523,566	2,451,409	2,403,792	2,522,861	2,548,130	2,545,726	2,596,081	2,522,665
Largest scaffold	745,183	750,526	1,241,341	254,917	1,561,578	745,183	544,931	1,241,354	470,345	470,661	674,517	1,241,350
N50	699,851	597,328	243,398	140,761	1,561,578	699,851	176,459	243,397	221,145	147,554	370,100	243,401
GC%	59.84	59.78	59.91	59.9	59.91	59.83	59.77	59.91	59.77	59.77	59.92	59.9
CDS	1,967	1,924	2,095	2,060	2,099	1,968	1,919	2,095	2,072	2,073	2,143	2,097
tRNA	57	58	59	64	59	57	58	59	59	59	58	60
rrn copy number	4	5	5	5	4	5	5	5	5	4	5	5

Supplementary Table 2: Conservation level of proteins and orthologous clusters into the 7 levels in this study.

Level	*B. moukalabense*	Human
0	all	all
1	all	not
2	all	variable
3	variable	all
4	variable	not
5	variable	variable
6	not	all
7	not	variable

Supplementary Table 3: COG category list.

J	Translation, ribosomal structure and biogenesis
A	RNA processing and modification
K	Transcription
L	Replication, recombination and repair
B	Chromatin structure and dynamics
D	Cell cycle control, cell division, chromosome partitioning
Y	Nuclear structure
V	Defense mechanisms
T	Signal transduction mechanisms
M	Cell wall/membrane/envelope biogenesis
N	Cell motility
Z	Cytoskeleton
W	Extracellular structures
U	Intracellular trafficking, secretion, and vesicular transport
O	Posttranslational modification, protein turnover, chaperones
X	Mobilome: prophages, transposons
C	Energy production and conversion
G	Carbohydrate transport and metabolism
E	Amino acid transport and metabolism
F	Nucleotide transport and metabolism
H	Coenzyme transport and metabolism
I	Lipid transport and metabolism
P	Inorganic ion transport and metabolism
Q	Secondary metabolites biosynthesis, transport and catabolism
R	General function prediction only
S	Function unknown

Supplementary Table 4: The number of ortholog clusters and proteins of the conservation levels broken down into the COG functional categories.

Ortholog clusters

level	J	A	K	L	B	D	Y	V	T	M	N	Z	W	U	O	X	C	G	E	F	H	I	P	Q	R	S
0	131	1	53	56	1	23	0	24	56	53	8	0	5	11	45	1	39	95	125	64	54	37	46	6	77	44
1&2	32	0	78	18	0	9	0	25	35	32	3	0	0	3	14	6	8	129	73	15	22	15	25	12	62	45
3&6	2	0	3	2	0	0	0	0	1	1	1	0	0	0	1	0	0	7	1	0	0	0	0	0	3	0
4	1	0	28	26	0	7	0	28	12	41	5	2	1	7	5	30	5	42	17	6	4	3	3	2	16	15
5	5	1	34	49	0	14	0	23	20	30	5	0	1	4	9	40	3	39	19	5	5	6	4	7	32	23
7	51	0	150	89	1	26	0	92	55	101	18	1	7	13	35	94	19	213	103	16	20	21	46	15	108	90

Proteins

level	J	A	K	L	B	D	Y	V	T	M	N	Z	W	U	O	X	C	G	E	F	H	I	P	Q	R	S
0	2490	19	1009	1064	19	430	0	455	1072	1039	134	0	94	208	876	19	741	1875	2433	1215	1045	760	874	123	1478	816
1&2	526	0	1254	290	12	170	0	405	572	573	43	0	0	54	249	102	119	2204	1212	208	357	244	447	177	1024	729
3&6	14	0	36	30	0	0	0	0	18	7	2	0	0	0	18	0	0	130	18	0	0	0	0	0	43	0
4	15	0	115	130	0	40	0	65	63	66	17	4	1	11	19	137	7	72	61	8	21	18	6	26	99	63
5	68	0	252	134	4	34	0	148	93	153	22	1	8	14	53	149	36	352	159	22	26	29	80	20	184	121
7	18	10	239	188	0	43	0	152	97	355	31	0	8	68	59	226	50	433	138	48	32	13	24	20	137	122

Supplementary Table 5: The list of the level 2 ortholog clusters within COG category G (carbohydrate metabolism and transport).

ortholog cluster ID	COG ID	COG description
1004	COG3345	Alpha-galactosidase
1013	COG3250	Beta-galactosidase/beta-glucuronidase
1017	COG2723	Beta-glucosidase/6-phospho-beta-glucosidase/beta-galactosidase
1028	COG0366	Glycosidase
1489	COG3537	Putative alpha-1,2-mannosidase
1809	COG3669	Alpha-L-fucosidase
1810	COG1472	Periplasmic beta-glucosidase and related glycosidases
1811	COG1501	Alpha-glucosidase, glycosyl hydrolase family GH31
1812	COG2730	Aryl-phospho-beta-D-glucosidase BglC, GH1 family
1814	COG1874	Beta-galactosidase GanA
1846	COG3250	Beta-galactosidase/beta-glucuronidase
1851	COG3507	Beta-xylosidase
1856	COG3507	Beta-xylosidase
1858	COG1501	Alpha-glucosidase, glycosyl hydrolase family GH31
1934	COG0366	Glycosidase
1977	COG3867	Arabinogalactan endo-1,4-beta-galactosidase
1982	COG3867	Arabinogalactan endo-1,4-beta-galactosidase
2039	COG0366	Glycosidase
2040	COG0366	Glycosidase
25160	COG1472	Periplasmic beta-glucosidase and related glycosidases
25259	COG3250	Beta-galactosidase/beta-glucuronidase
25306	COG3250	Beta-galactosidase/beta-glucuronidase
25332	COG3345	Alpha-galactosidase
25540	COG1874	Beta-galactosidase GanA
25547	COG2730	Aryl-phospho-beta-D-glucosidase BglC, GH1 family
25548	COG3507	Beta-xylosidase
25694	COG0366	Glycosidase
26000	COG3867	Arabinogalactan endo-1,4-beta-galactosidase
26072	COG1472	Periplasmic beta-glucosidase and related glycosidases
26097	COG1472	Periplasmic beta-glucosidase and related glycosidases
26271	COG3534	Alpha-L-arabinofuranosidase
63	COG1501	Alpha-glucosidase, glycosyl hydrolase family GH31
85	COG0366	Glycosidase
912	COG3507	Beta-xylosidase
921	COG3345	Alpha-galactosidase
927	COG1472	Periplasmic beta-glucosidase and related glycosidases
937	COG1472	Periplasmic beta-glucosidase and related glycosidases
947	COG1472	Periplasmic beta-glucosidase and related glycosidases
1856	COG3507	Beta-xylosidase
947	COG1472	Periplasmic beta-glucosidase and related glycosidases
1856	COG3507	Beta-xylosidase

Supplementary Table 6: The list of level 1 ortholog clusters with the COG annotation.

ortholog cluster ID	COG ID	COG category	COG description
32	COG1254	C	Acylphosphatase
51	COG2207	K	AraC-type DNA-binding domain and AraC-containing proteins
51	COG1917	R	Cupin domain protein related to quercetin dioxygenase
129	COG4292	S	Low temperature requirement protein LtrA (function unknown)
131	COG1174	E	ABC-type proline/glycine betaine transport system, permease component
131	COG1732	M	Periplasmic glycine betaine/choline-binding (lipo)protein of an ABC-type transport system (osmoprotectant binding protein)
132	COG1125	E	ABC-type proline/glycine betaine transport system, ATPase component
133	COG0604	CR	NADPH:quinone reductase or related Zn-dependent oxidoreductase
136	COG3548	S	Uncharacterized membrane protein
137	COG1154	HI	Deoxyxylulose-5-phosphate synthase
138	COG0667	R	Predicted oxidoreductase (related to aryl-alcohol dehydrogenase)
139	COG0300	R	Short-chain dehydrogenase
273	COG1132	V	ABC-type multidrug transport system, ATPase and permease component
274	COG1132	V	ABC-type multidrug transport system, ATPase and permease component
284	COG1846	K	DNA-binding transcriptional regulator, MarR family
285	COG1131	V	ABC-type multidrug transport system, ATPase component
286	COG0842	V	ABC-type multidrug transport system, permease component
474	COG1972	F	Nucleoside permease NupC
475	COG0213	F	Thymidine phosphorylase
477	COG1015	G	Phosphopentomutase
478	COG0274	F	Deoxyribose-phosphate aldolase
886	COG3764	M	Sortase (surface protein transpeptidase)
888	COG4932	S	Uncharacterized surface anchored protein
889	COG3764	M	Sortase (surface protein transpeptidase)
925	COG2814	G	Predicted arabinose efflux permease, MFS family
928	COG2211	G	Na+/melibiose symporter or related transporter
929	COG3408	G	Glycogen debranching enzyme (alpha-1,6-glucosidase)
1074	COG2110	J	O-acetyl-ADP-ribose deacetylase (regulator of RNase III), contains Macro domain
1075	COG0846	O	NAD-dependent protein deacetylase, SIR2 family
1086	COG2814	G	Predicted arabinose efflux permease, MFS family
1124	COG2268	S	Uncharacterized membrane protein YqiK, contains Band7/PHB/SPFH domain
1218	COG0457	R	Tetratricopeptide (TPR) repeat
1464	COG1533	L	DNA repair photolyase
1465	COG0778	C	Nitroreductase
1466	COG1846	K	DNA-binding transcriptional regulator, MarR family
1467	COG4886	K	Leucine-rich repeat (LRR) protein
1720	COG0222	J	Ribosomal protein L7/L12
1792	COG4126	E	Asp/Glu/hydantoin racemase
1793	COG1953	FH	Cytosine/uracil/thiamine/allantoin permease
1795	COG0044	F	Dihydroorotase or related cyclic amidohydrolase
1796	COG0624	E	Acetylornithine deacetylase/Succinyl-diaminopimelate desuccinylase or related deacylase
1797	COG0075	EF	Archaeal aspartate aminotransferase or a related aminotransferase, includes purine catabolism protein PucG
1798	COG1737	K	DNA-binding transcriptional regulator, MurR/RpiR family, contains HTH and SIS domains
1818	COG0531	E	Amino acid transporter
2055	COG4367	S	Uncharacterized protein
2060	COG2977	Q	4’-phosphopantetheinyl transferase EntD (siderophore biosynthesis)
2063	COG3321	Q	Acyl transferase domain in polyketide synthase (PKS) enzymes
2066	COG3560	R	Fatty acid repression mutant protein (predicted oxidoreductase)

Supplementary Table 7: The list of level 2 ortholog clusters with the COG annotation.

ortholog cluster ID	COG ID	COG category	COG description
2	COG0281	C	Malic enzyme
9	COG2755	E	Lysophospholipase L1 or related esterase
17	COG2071	E	Gamma-glutamyl-gamma-aminobutyrate hydrolase PuuD (putrescine degradation), contains GATase1-like domain
27	COG1357	S	Uncharacterized protein YjbI, contains pentapeptide repeats
52	COG1280	E	Threonine/homoserine/homoserine lactone efflux protein
53	COG0531	E	Amino acid transporter
53	COG1454	C	Alcohol dehydrogenase, class IV
54	COG1102	F	Cytidylate kinase
55	COG4992	E	Acetylornithine/succinyldiaminopimelate/putrescine aminotransferase
56	COG2508	K	DNA-binding transcriptional regulator, PucR family
60	COG0667	R	Predicted oxidoreductase (related to aryl-alcohol dehydrogenase)
61	COG0534	V	Na+-driven multidrug efflux pump
62	COG1609	K	DNA-binding transcriptional regulator, LacI/PurR family
63	COG1501	G	Alpha-glucosidase, glycosyl hydrolase family GH31
85	COG0366	G	Glycosidase
85	COG1523	G	Pullulanase/glycogen debranching enzyme
85	COG3266	D	Cell division protein DamX, binds to the septal ring, contains C-terminal SPOR domain
140	COG0582	LX	Integrase
172	COG0494	V	8-oxo-dGTP pyrophosphatase MutT and related house-cleaning NTP pyrophosphohydrolases, NUDIX family
187	COG1040	R	Predicted amidophosphoribosyltransferases
194	COG1316	M	Anionic cell wall polymer biosynthesis enzyme, LytR-Cps2A-Psr (LCP) family
271	COG2879	S	Uncharacterized short protein YbdD, DUF466 family
272	COG1966	T	Carbon starvation protein CstA
278	COG0656	Q	Aldo/keto reductase, related to diketogulonate reductase
279	COG0110	R	Acetyltransferase (isoleucine patch superfamily)
280	COG0583	K	DNA-binding transcriptional regulator, LysR family
283	COG0350	L	O6-methylguanine-DNA–protein-cysteine methyltransferase
288	COG1985	H	Pyrimidine reductase, riboflavin biosynthesis
293	COG3340	E	Peptidase E
307	COG0583	K	DNA-binding transcriptional regulator, LysR family
310	COG0697	GER	Permease of the drug/metabolite transporter (DMT) superfamily
312	COG4194	S	Uncharacterized membrane protein
313	COG1725	K	DNA-binding transcriptional regulator YhcF, GntR family
314	COG1510	K	DNA-binding transcriptional regulator GbsR, MarR family
314	COG3177	K	Fic family protein
412	COG1402	HQ	Creatinine amidohydrolase/Fe(II)-dependent formamide hydrolase involved in riboflavin and F420 biosynthesis
413	COG0477	GEPR	MFS family permease
414	COG2508	K	DNA-binding transcriptional regulator, PucR family
415	COG0402	FR	Cytosine/adenosine deaminase or related metal-dependent hydrolase
448	COG2230	I	Cyclopropane fatty-acyl-phospholipid synthase and related methyltransferases
476	COG2390	K	DNA-binding transcriptional regulator LsrR, DeoR family
488	COG2963	X	Transposase and inactivated derivatives
508	COG3238	S	Uncharacterized membrane protein YdcZ, DUF606 family
511	COG0232	F	dGTP triphosphohydrolase
555	COG2211	G	Na+/melibiose symporter or related transporter
557	COG1609	K	DNA-binding transcriptional regulator, LacI/PurR family
596	COG4214	G	ABC-type xylose transport system, permease component
597	COG1129	G	ABC-type sugar transport system, ATPase component
598	COG4213	G	ABC-type xylose transport system, periplasmic component
623	COG1131	V	ABC-type multidrug transport system, ATPase component
636	COG0791	M	Cell wall-associated hydrolase, NlpC family
639	COG1482	G	Mannose-6-phosphate isomerase, class I
656	COG3290	T	Sensor histidine kinase regulating citrate/malate metabolism
657	COG3279	KT	DNA-binding response regulator, LytR/AlgR family
659	COG1132	V	ABC-type multidrug transport system, ATPase and permease component
660	COG1132	V	ABC-type multidrug transport system, ATPase and permease component
661	COG1073	T	Fermentation-respiration switch protein FrsA, has esterase activity, DUF1100 family
663	COG1983	KT	Phage shock protein PspC (stress-responsive transcriptional regulator)
680	COG3279	KT	DNA-binding response regulator, LytR/AlgR family
681	COG3290	T	Sensor histidine kinase regulating citrate/malate metabolism
685	COG1476	K	DNA-binding transcriptional regulator, XRE-family HTH domain
690	COG0499	H	S-adenosylhomocysteine hydrolase
691	COG0402	FR	Cytosine/adenosine deaminase or related metal-dependent hydrolase
692	COG3542	R	Predicted sugar epimerase, cupin superfamily
693	COG0834	ET	ABC-type amino acid transport/signal transduction system, periplasmic component/domain
694	COG0765	E	ABC-type amino acid transport system, permease component
695	COG1126	E	ABC-type polar amino acid transport system, ATPase component
699	COG0532	J	Translation initiation factor IF-2, a GTPase
699	COG3266	D	Cell division protein DamX, binds to the septal ring, contains C-terminal SPOR domain
702	COG1305	O	Transglutaminase-like enzyme, putative cysteine protease
703	COG2307	S	Uncharacterized conserved protein, Alpha-E superfamily
703	COG2308	S	Uncharacterized conserved protein, circularly permuted ATPgrasp superfamily
709	COG0257	J	Ribosomal protein L36
749	COG5006	E	Threonine/homoserine efflux transporter RhtA

Table continues

Table continued

ortholog cluster ID	COG ID	COG category	COG description
785	COG1167	KE	DNA-binding transcriptional regulator, MocR family, contains an aminotransferase domain
786	COG1279	E	Arginine exporter protein ArgO
803	COG0080	J	Ribosomal protein L11
811	COG1530	J	Ribonuclease G or E
820	COG0836	M	Mannose-1-phosphate guanylyltransferase
826	COG0667	R	Predicted oxidoreductase (related to aryl-alcohol dehydrogenase)
831	COG0333	J	Ribosomal protein L32
843	COG1027	E	Aspartate ammonia-lyase
848	COG0457	R	Tetratricopeptide (TPR) repeat
850	COG1708	R	Predicted nucleotidyltransferase
851	COG1075	I	Triacylglycerol esterase/lipase EstA, alpha/beta hydrolase fold
859	COG4905	S	Uncharacterized membrane protein
866	COG2963	X	Transposase and inactivated derivatives
873	COG2972	T	Sensor histidine kinase YesM
874	COG3947	TK	Two-component response regulator, SAPR family, consists of REC, wHTH and BTAD domains
880	COG3152	S	Uncharacterized membrane protein YhaH, DUF805 family
902	COG0562	M	UDP-galactopyranose mutase
912	COG3507	G	Beta-xylosidase
921	COG3345	G	Alpha-galactosidase
922	COG0662	G	Mannose-6-phosphate isomerase, cupin superfamily
922	COG2207	K	AraC-type DNA-binding domain and AraC-containing proteins
927	COG1472	G	Periplasmic beta-glucosidase and related glycosidases
930	COG3279	KT	DNA-binding response regulator, LytR/AlgR family
935	COG3290	T	Sensor histidine kinase regulating citrate/malate metabolism
936	COG3279	KT	DNA-binding response regulator, LytR/AlgR family
937	COG1472	G	Periplasmic beta-glucosidase and related glycosidases
946	COG2211	G	Na+/melibiose symporter or related transporter
947	COG1472	G	Periplasmic beta-glucosidase and related glycosidases
947	COG2890	J	Methylase of polypeptide chain release factors
948	COG1309	K	DNA-binding transcriptional regulator, AcrR family
962	COG0475	P	Kef-type K+ transport system, membrane component KefB
970	COG5434	G	Polygalacturonase
981	COG2211	G	Na+/melibiose symporter or related transporter
982	COG0174	E	Glutamine synthetase
983	COG2814	G	Predicted arabinose efflux permease, MFS family
985	COG2211	G	Na+/melibiose symporter or related transporter
987	COG1672	R	Predicted ATPase, archaeal AAA+ ATPase superfamily
1004	COG3345	G	Alpha-galactosidase
1006	COG5578	S	Uncharacterized membrane protein YesL
1012	COG1609	K	DNA-binding transcriptional regulator, LacI/PurR family
1013	COG3250	G	Beta-galactosidase/beta-glucuronidase
1014	COG0395	G	ABC-type glycerol-3-phosphate transport system, permease component
1015	COG1175	G	ABC-type sugar transport system, permease component
1016	COG1653	G	ABC-type glycerol-3-phosphate transport system, periplasmic component
1017	COG2723	G	Beta-glucosidase/6-phospho-beta-glucosidase/beta-galactosidase
1018	COG2755	E	Lysophospholipase L1 or related esterase
1024	COG2211	G	Na+/melibiose symporter or related transporter
1025	COG0395	G	ABC-type glycerol-3-phosphate transport system, permease component
1026	COG1175	G	ABC-type sugar transport system, permease component
1027	COG2182	G	Maltose-binding periplasmic protein MalE
1028	COG0366	G	Glycosidase
1029	COG5578	S	Uncharacterized membrane protein YesL
1032	COG4770	I	Acetyl/propionyl-CoA carboxylase, alpha subunit
1033	COG1984	E	Allophanate hydrolase subunit 2
1033	COG2049	E	Allophanate hydrolase subunit 1
1034	COG1540	R	Lactam utilization protein B (function unknown)
1035	COG4336	S	Uncharacterized protein YcsI, UPF0317 family
1036	COG0115	EH	Branched-chain amino acid aminotransferase/4-amino-4-deoxychorismate lyase
1038	COG0683	E	ABC-type branched-chain amino acid transport system, periplasmic component
1040	COG0410	E	ABC-type branched-chain amino acid transport system, ATPase component
1041	COG0411	E	ABC-type branched-chain amino acid transport system, ATPase component
1042	COG4177	E	ABC-type branched-chain amino acid transport system, permease component
1043	COG0559	E	Branched-chain amino acid ABC-type transport system, permease component
1045	COG0239	DP	Fluoride ion exporter CrcB/FEX, affects chromosome condensation
1048	COG0726	GM	Peptidoglycan/xylan/chitin deacetylase, PgdA/CDA1 family
1050	COG5340	V	Transcriptional regulator, predicted component of viral defense system
1053	COG0778	C	Nitroreductase
1065	COG2367	V	Beta-lactamase class A
1067	COG1113	E	L-asparagine transporter and related permeases
1072	COG1733	K	DNA-binding transcriptional regulator, HxlR family
1073	COG2910	R	Putative NADH-flavin reductase
1082	COG1102	F	Cytidylate kinase
1082	COG2364	S	Uncharacterized membrane protein YczE
1142	COG0438	M	Glycosyltransferase involved in cell wall bisynthesis
1143	COG1560	I	Lauroyl/myristoyl acyltransferase
1180	COG1555	L	DNA uptake protein ComE and related DNA-binding proteins

Table continues

Table continued

ortholog cluster ID	COG ID	COG category	COG description
1187	COG3947	TK	Two-component response regulator, SAPR family, consists of REC, wHTH and BTAD domains
1209	COG0517	T	CBS domain
1220	COG4905	S	Uncharacterized membrane protein
1235	COG0577	V	ABC-type antimicrobial peptide transport system, permease component
1266	COG1228	Q	Imidazolonepropionase or related amidohydrolase
1275	COG0534	V	Na+-driven multidrug efflux pump
1278	COG3189	S	Uncharacterized conserved protein YeaO, DUF488 family
1282	COG3824	O	Predicted Zn-dependent protease, minimal metalloprotease (MMP)-like domain
1283	COG1490	J	D-Tyr-tRNAtyr deacylase
1284	COG1816	F	Adenosine deaminase
1289	COG2110	J	O-acetyl-ADP-ribose deacetylase (regulator of RNase III), contains Macro domain
1290	COG2021	E	Homoserine acetyltransferase
1291	COG0110	R	Acetyltransferase (isoleucine patch superfamily)
1293	COG1917	R	Cupin domain protein related to quercetin dioxygenase
1293	COG2207	K	AraC-type DNA-binding domain and AraC-containing proteins
1294	COG0507	L	ATP-dependent exoDNAse (exonuclease V), alpha subunit, helicase superfamily I
1294	COG3410	S	Uncharacterized protein, DUF2075 family
1295	COG1694	V	NTP pyrophosphatase, house-cleaning of non-canonical NTPs
1297	COG4842	S	Uncharacterized conserved protein YukE
1298	COG4842	S	Uncharacterized conserved protein YukE
1300	COG1404	O	Serine protease, subtilisin family
1307	COG2271	G	Sugar phosphate permease
1325	COG4585	T	Signal transduction histidine kinase
1326	COG2197	TK	DNA-binding response regulator, NarL/FixJ family, contains REC and HTH domains
1327	COG4826	O	Serine protease inhibitor
1350	COG2094	L	3-methyladenine DNA glycosylase Mpg
1359	COG2814	G	Predicted arabinose efflux permease, MFS family
1360	COG1917	R	Cupin domain protein related to quercetin dioxygenase
1360	COG2207	K	AraC-type DNA-binding domain and AraC-containing proteins
1361	COG0477	GEPR	MFS family permease
1362	COG2017	G	Galactose mutarotase or related enzyme
1363	COG0524	G	Sugar or nucleoside kinase, ribokinase family
1364	COG3822	G	D-lyxose ketol-isomerase
1365	COG1349	KG	DNA-binding transcriptional regulator of sugar metabolism, DeoR/GlpR family
1366	COG0036	G	Pentose-5-phosphate-3-epimerase
1367	COG0794	GM	D-arabinose 5-phosphate isomerase GutQ
1370	COG1846	K	DNA-binding transcriptional regulator, MarR family
1371	COG0110	R	Acetyltransferase (isoleucine patch superfamily)
1374	COG0673	R	Predicted dehydrogenase
1375	COG2814	G	Predicted arabinose efflux permease, MFS family
1377	COG0789	K	DNA-binding transcriptional regulator, MerR family
1379	COG0477	GEPR	MFS family permease
1380	COG1846	K	DNA-binding transcriptional regulator, MarR family
1381	COG2211	G	Na+/melibiose symporter or related transporter
1382	COG5512	R	Predicted nucleic acid-binding protein, contains Zn-ribbon domain (includes truncated derivatives)
1383	COG1309	K	DNA-binding transcriptional regulator, AcrR family
1384	COG2211	G	Na+/melibiose symporter or related transporter
1387	COG1309	K	DNA-binding transcriptional regulator, AcrR family
1389	COG3290	T	Sensor histidine kinase regulating citrate/malate metabolism
1390	COG3279	KT	DNA-binding response regulator, LytR/AlgR family
1394	COG0702	R	Uncharacterized conserved protein YbjT, contains NAD(P)-binding and DUF2867 domains
1408	COG0346	Q	Catechol 2,3-dioxygenase or other lactoylglutathione lyase family enzyme
1409	COG0738	G	Fucose permease
1415	COG0494	V	8-oxo-dGTP pyrophosphatase MutT and related house-cleaning NTP pyrophosphohydrolases, NUDIX family
1416	COG1061	KL	Superfamily II DNA or RNA helicase
1416	COG3886	L	HKD family nuclease
1440	COG2190	G	Phosphotransferase system IIA component
1468	COG1804	I	Crotonobetainyl-CoA:carnitine CoA-transferase CaiB and related acyl-CoA transferases
1470	COG0679	R	Predicted permease
1471	COG0028	EH	Acetolactate synthase large subunit or other thiamine pyrophosphate-requiring enzyme
1476	COG4585	T	Signal transduction histidine kinase
1487	COG1158	K	Transcription termination factor Rho
1489	COG3537	G	Putative alpha-1,2-mannosidase
1503	COG2304	R	Secreted protein containing bacterial Ig-like domain and vWFA domain
1508	COG5479	S	Uncharacterized conserved protein, contains LGFP repeats
1510	COG4932	S	Uncharacterized surface anchored protein
1513	COG0629	L	Single-stranded DNA-binding protein
1521	COG1216	G	Glycosyltransferase, GT2 family
1532	COG1714	S	Uncharacterized membrane protein YckC, RDD family
1532	COG1716	T	Forkhead associated (FHA) domain, binds pSer, pThr, pTyr
1533	COG1674	D	DNA segregation ATPase FtsK/SpoIIIE and related proteins
1536	COG2814	G	Predicted arabinose efflux permease, MFS family
1543	COG1388	M	LysM repeat
1545	COG0111	HR	Phosphoglycerate dehydrogenase or related dehydrogenase
1559	COG2942	G	Mannose or cellobiose epimerase, N-acyl-D-glucosamine 2-epimerase family
1615	COG3538	S	Meiotically up-regulated gene 157 (Mug157) protein (function unknown)

Table continues

Table continued

ortholog cluster ID	COG ID	COG category	COG description
1618	COG0747	E	ABC-type transport system, periplasmic component
1619	COG0601	EP	ABC-type dipeptide/oligopeptide/nickel transport system, permease component
1620	COG1173	EP	ABC-type dipeptide/oligopeptide/nickel transport system, permease component
1621	COG4172	Q	ABC-type microcin C transport system, duplicated ATPase component YejF
1625	COG1794	M	Aspartate/glutamate racemase
1626	COG3919	R	Predicted ATP-dependent carboligase, ATP-grasp superfamily
1636	COG0367	E	Asparagine synthetase B (glutamine-hydrolyzing)
1643	COG0379	H	Quinolinate synthase
1647	COG0477	GEPR	MFS family permease
1653	COG0577	V	ABC-type antimicrobial peptide transport system, permease component
1653	COG4591	M	ABC-type transport system, involved in lipoprotein release, permease component
1654	COG1136	M	ABC-type lipoprotein export system, ATPase component
1673	COG4585	T	Signal transduction histidine kinase
1674	COG2197	TK	DNA-binding response regulator, NarL/FixJ family, contains REC and HTH domains
1681	COG2184	T	Fido, protein-threonine AMPylation domain
1682	COG4124	G	Beta-mannanase
1701	COG2205	T	K+-sensing histidine kinase KdpD
1701	COG2770	T	HAMP domain
1728	COG3835	KT	Sugar diacid utilization regulator
1729	COG2610	GR	H+/gluconate symporter or related permease
1736	COG0847	L	DNA polymerase III, epsilon subunit or related 3'-5' exonuclease
1739	COG1250	I	3-hydroxyacyl-CoA dehydrogenase
1758	COG1216	G	Glycosyltransferase, GT2 family
1762	COG2814	G	Predicted arabinose efflux permease, MFS family
1763	COG1940	KG	Sugar kinase of the NBD/HSP70 family, may contain an N-terminal HTH domain
1764	COG0036	G	Pentose-5-phosphate-3-epimerase
1765	COG1940	KG	Sugar kinase of the NBD/HSP70 family, may contain an N-terminal HTH domain
1766	COG2814	G	Predicted arabinose efflux permease, MFS family
1777	COG0631	T	Serine/threonine protein phosphatase PrpC
1787	COG0454	KR	N-acetyltransferase, GNAT superfamily (includes histone acetyltransferase HPA2)
1790	COG1937	K	DNA-binding transcriptional regulator, FrmR family
1794	COG0154	J	Asp-tRNAAsn/Glu-tRNAGln amidotransferase A subunit or related amidase
1799	COG2807	P	Cyanate permease
1805	COG0531	E	Amino acid transporter
1806	COG0076	E	Glutamate or tyrosine decarboxylase or a related PLP-dependent protein
1809	COG3669	G	Alpha-L-fucosidase
1810	COG1472	G	Periplasmic beta-glucosidase and related glycosidases
1811	COG1501	G	Alpha-glucosidase, glycosyl hydrolase family GH31
1812	COG2730	G	Aryl-phospho-beta-D-glucosidase BglC, GH1 family
1814	COG1874	G	Beta-galactosidase GanA
1815	COG1940	KG	Sugar kinase of the NBD/HSP70 family, may contain an N-terminal HTH domain
1816	COG2508	K	DNA-binding transcriptional regulator, PucR family
1819	COG0160	E	4-aminobutyrate aminotransferase or related aminotransferase
1820	COG1454	C	Alcohol dehydrogenase, class IV
1829	COG0657	I	Acetyl esterase/lipase
1830	COG3459	G	Cellobiose phosphorylase
1834	COG4124	G	Beta-mannanase
1835	COG1928	O	Dolichyl-phosphate-mannose–protein O-mannosyl transferase
1842	COG2814	G	Predicted arabinose efflux permease, MFS family
1846	COG3250	G	Beta-galactosidase/beta-glucuronidase
1847	COG1609	K	DNA-binding transcriptional regulator, LacI/PurR family
1848	COG1653	G	ABC-type glycerol-3-phosphate transport system, periplasmic component
1850	COG0395	G	ABC-type glycerol-3-phosphate transport system, permease component
1851	COG3507	G	Beta-xylosidase
1852	COG5578	S	Uncharacterized membrane protein YesL
1856	COG1621	G	Sucrose-6-phosphate hydrolase SacC, GH32 family
1856	COG3507	G	Beta-xylosidase
1857	COG1609	K	DNA-binding transcriptional regulator, LacI/PurR family
1858	COG1501	G	Alpha-glucosidase, glycosyl hydrolase family GH31
1860	COG2211	G	Na+/melibiose symporter or related transporter
1871	COG3583	S	Uncharacterized conserved protein YabE, contains G5 and tandem DUF348 domains
1878	COG0728	M	Peptidoglycan biosynthesis protein MviN/MurJ, putative lipid II flippase
1879	COG0028	EH	Acetolactate synthase large subunit or other thiamine pyrophosphate-requiring enzyme
1897	COG0783	PV	DNA-binding ferritin-like protein (oxidative damage protectant)
1934	COG0366	G	Glycosidase
1935	COG2182	G	Maltose-binding periplasmic protein MalE
1936	COG1175	G	ABC-type sugar transport system, permease component
1937	COG3833	G	ABC-type maltose transport system, permease component
1938	COG1523	G	Pullulanase/glycogen debranching enzyme
1939	COG1011	H	FMN phosphatase YigB, HAD superfamily
1942	COG0730	S	Uncharacterized membrane protein YfcA
1953	COG2801	X	Transposase InsO and inactivated derivatives
1953	COG2963	X	Transposase and inactivated derivatives
1957	COG2182	G	Maltose-binding periplasmic protein MalE
1961	COG5578	S	Uncharacterized membrane protein YesL
1962	COG4209	G	ABC-type polysaccharide transport system, permease component
1963	COG0395	G	ABC-type glycerol-3-phosphate transport system, permease component

Table continued

ortholog cluster ID	COG ID	COG category	COG description
1964	COG1653	G	ABC-type glycerol-3-phosphate transport system, periplasmic component
1974	COG2755	E	Lysophospholipase L1 or related esterase
1976	COG2351	F	5-hydroxyisourate hydrolase (purine catabolism), transthyretin-related family
1976	COG3391	R	DNA-binding beta-propeller fold protein YncE
1976	COG4886	K	Leucine-rich repeat (LRR) protein
1977	COG3867	G	Arabinogalactan endo-1,4-beta-galactosidase
1977	COG5183	O	E3 ubiquitin-protein ligase DOA10
1977	COG5263	G	Glucan-binding domain (YG repeat)
1982	COG3867	G	Arabinogalactan endo-1,4-beta-galactosidase
1992	COG1216	G	Glycosyltransferase, GT2 family
1993	COG1215	N	Glycosyltransferase, catalytic subunit of cellulose synthase and poly-beta-1,6-N-acetylglucosamine synthase
1993	COG1216	G	Glycosyltransferase, GT2 family
1994	COG1835	M	Peptidoglycan/LPS O-acetylase OafA/YrhL, contains acyltransferase and SGNH-hydrolase domains
1997	COG0463	M	Glycosyltransferase involved in cell wall bisynthesis
2018	COG1209	M	dTDP-glucose pyrophosphorylase
2019	COG1091	M	dTDP-4-dehydrorhamnose reductase
2019	COG1898	M	dTDP-4-dehydrorhamnose 3,5-epimerase or related enzyme
2035	COG1609	K	DNA-binding transcriptional regulator, LacI/PurR family
2036	COG1653	G	ABC-type glycerol-3-phosphate transport system, periplasmic component
2037	COG1175	G	ABC-type sugar transport system, permease component
2038	COG0395	G	ABC-type glycerol-3-phosphate transport system, permease component
2039	COG0366	G	Glycosidase
2040	COG0366	G	Glycosidase
2048	COG3290	T	Sensor histidine kinase regulating citrate/malate metabolism
2051	COG0436	E	Aspartate/methionine/tyrosine aminotransferase
2061	COG1309	K	DNA-binding transcriptional regulator, AcrR family
2065	COG0477	GEPR	MFS family permease
2074	COG0394	T	Protein-tyrosine-phosphatase
2078	COG0803	P	ABC-type Zn uptake system ZnuABC, Zn-binding component ZnuA
2078	COG3443	P	Periplasmic Zn/Cd-binding protein ZinT
2080	COG2801	X	Transposase InsO and inactivated derivatives
2080	COG2963	X	Transposase and inactivated derivatives
2081	COG2801	X	Transposase InsO and inactivated derivatives
2081	COG2963	X	Transposase and inactivated derivatives
2088	COG0456	J	Ribosomal protein S18 acetylase RimI and related acetyltransferases
2088	COG1670	JO	Protein N-acetyltransferase, RimJ/RimL family
2092	COG1672	R	Predicted ATPase, archaeal AAA+ ATPase superfamily
2095	COG2852	L	Very-short-patch-repair endonuclease
24538	COG1643	J	HrpA-like RNA helicase
24543	COG1974	KT	SOS-response transcriptional repressor LexA (RecA-mediated autopeptidase)
24544	COG1327	K	Transcriptional regulator NrdR, contains Zn-ribbon and ATP-cone domains
24560	COG1589	D	Cell division septal protein FtsQ
24579	COG0290	J	Translation initiation factor IF-3
24586	COG0029	H	Aspartate oxidase
24587	COG0157	H	Nicotinate-nucleotide pyrophosphorylase
24588	COG1104	E	Cysteine sulfinate desulfinase/cysteine desulfurase or related enzyme
24598	COG0444	EP	ABC-type dipeptide/oligopeptide/nickel transport system, ATPase component
24598	COG4172	Q	ABC-type microcin C transport system, duplicated ATPase component YejF
24607	COG0110	R	Acetyltransferase (isoleucine patch superfamily)
24608	COG1917	R	Cupin domain protein related to quercetin dioxygenase
24608	COG2207	K	AraC-type DNA-binding domain and AraC-containing proteins
24616	COG0697	GER	Permease of the drug/metabolite transporter (DMT) superfamily
24627	COG2137	O	SOS response regulatory protein OraA/RecX, interacts with RecA
24646	COG0568	K	DNA-directed RNA polymerase, sigma subunit (sigma70/sigma32)
24657	COG0652	O	Peptidyl-prolyl cis-trans isomerase (rotamase) - cyclophilin family
24669	COG1135	E	ABC-type methionine transport system, ATPase component
24670	COG1464	P	ABC-type metal ion transport system, periplasmic component/surface antigen
24679	COG0349	J	Ribonuclease D
24681	COG1434	R	Uncharacterized SAM-binding protein YcdF, DUF218 family
24683	COG1917	R	Cupin domain protein related to quercetin dioxygenase
24701	COG2356	L	Endonuclease I
24708	COG0819	H	Thiaminase
24721	COG1122	PR	Energy-coupling factor transporter ATP-binding protein EcfA2
24722	COG0619	H	Energy-coupling factor transporter transmembrane protein EcfT
24730	COG1611	R	Predicted Rossmann fold nucleotide-binding protein
24736	COG1136	M	ABC-type lipoprotein export system, ATPase component
24740	COG0016	J	Phenylalanyl-tRNA synthetase alpha subunit
24741	COG0072	J	Phenylalanyl-tRNA synthetase beta subunit
24741	COG0073	J	tRNA-binding EMAP/Myf domain
24743	COG0002	E	N-acetyl-gamma-glutamylphosphate reductase
24746	COG4992	E	Acetylornithine/succinyldiaminopimelate/putrescine aminotransferase
24748	COG1438	K	Arginine repressor
24757	COG1189	J	Predicted rRNA methylase YqxC, contains S4 and FtsJ domains
24767	COG1725	K	DNA-binding transcriptional regulator YhcF, GntR family
24771	COG3467	V	Nitroimidazol reductase NimA or a related FMN-containing flavoprotein, pyridoxamine 5’-phosphate oxidase superfamily
24779	COG1028	IQR	NAD(P)-dependent dehydrogenase, short-chain alcohol dehydrogenase family
24783	COG2814	G	Predicted arabinose efflux permease, MFS family

Table continued

ortholog cluster ID	COG ID	COG category	COG description
24786	COG0112	E	Glycine/serine hydroxymethyltransferase
24804	COG0133	E	Tryptophan synthase beta chain
24804	COG0134	E	Indole-3-glycerol phosphate synthase
24807	COG0036	G	Pentose-5-phosphate-3-epimerase
24809	COG0040	E	ATP phosphoribosyltransferase
24815	COG0558	I	Phosphatidylglycerophosphate synthase
24830	COG4284	G	UDP-N-acetylglucosamine pyrophosphorylase
24855	COG1481	K	DNA-binding transcriptional regulator WhiA, involved in cell division
24858	COG0322	L	Excinuclease UvrABC, nuclease subunit
24867	COG1917	R	Cupin domain protein related to quercetin dioxygenase
24868	COG4767	V	Glycopeptide antibiotics resistance protein
24880	COG0053	P	Divalent metal cation (Fe/Co/Zn/Cd) transporter
24881	COG0599	R	Uncharacterized conserved protein YurZ, alkylhydroperoxidase/carboxymuconolactone decarboxylase family
24883	COG0789	K	DNA-binding transcriptional regulator, MerR family
24890	COG0802	J	tRNA A37 threonylcarbamoyladenosine biosynthesis protein TsaE
24897	COG0406	G	Broad specificity phosphatase PhoE
24900	COG4932	S	Uncharacterized surface anchored protein
24916	COG0739	M	Murein DD-endopeptidase MepM and murein hydrolase activator NlpD, contain LysM domain
24925	COG0620	E	Methionine synthase II (cobalamin-independent)
24929	COG0583	K	DNA-binding transcriptional regulator, LysR family
25012	COG0239	DP	Fluoride ion exporter CrcB/FEX, affects chromosome condensation
25013	COG0239	DP	Fluoride ion exporter CrcB/FEX, affects chromosome condensation
25063	COG1950	S	Uncharacterized membrane protein YvlD, DUF360 family
25081	COG0534	V	Na+-driven multidrug efflux pump
25113	COG0258	L	5'-3' exonuclease
25113	COG0749	L	DNA polymerase I - 3'-5' exonuclease and polymerase domains
25141	COG1316	M	Anionic cell wall polymer biosynthesis enzyme, LytR-Cps2A-Psr (LCP) family
25154	COG2919	D	Cell division protein FtsB
25160	COG1472	G	Periplasmic beta-glucosidase and related glycosidases
25163	COG2249	R	Putative NADPH-quinone reductase (modulator of drug activity B)
25167	COG0300	R	Short-chain dehydrogenase
25168	COG0364	G	Glucose-6-phosphate 1-dehydrogenase
25183	COG0627	V	S-formylglutathione hydrolase FrmB
25194	COG2207	K	AraC-type DNA-binding domain and AraC-containing proteins
25259	COG3250	G	Beta-galactosidase/beta-glucuronidase
25273	COG1785	PR	Alkaline phosphatase
25306	COG3250	G	Beta-galactosidase/beta-glucuronidase
25332	COG3345	G	Alpha-galactosidase
25333	COG0084	N	Tat protein secretion system quality control protein TatD (DNase activity)
25337	COG1136	M	ABC-type lipoprotein export system, ATPase component
25352	COG2217	P	Cation transport ATPase
25352	COG3350	S	Uncharacterized conserved protein, YHS domain
25352	COG4633	R	Plastocyanin domain containing protein
25365	COG1522	K	DNA-binding transcriptional regulator, Lrp family
25388	COG0328	L	Ribonuclease HI
25420	COG0791	M	Cell wall-associated hydrolase, NlpC family
25429	COG0220	J	tRNA G46 methylase TrmB
25446	COG1680	V	CubicO group peptidase, beta-lactamase class C family
25450	COG1574	R	Predicted amidohydrolase YtcJ
25451	COG2873	E	O-acetylhomoserine/O-acetylserine sulfhydrylase, pyridoxal phosphate-dependent
25479	COG0411	E	ABC-type branched-chain amino acid transport system, ATPase component
25485	COG0561	HR	Hydroxymethylpyrimidine pyrophosphatase and other HAD family phosphatases
25490	COG0629	L	Single-stranded DNA-binding protein
25491	COG3590	O	Predicted metalloendopeptidase
25508	COG0834	ET	ABC-type amino acid transport/signal transduction system, periplasmic component/domain
25509	COG0765	E	ABC-type amino acid transport system, permease component
25515	COG4279	S	Uncharacterized conserved protein, contains Zn finger domain
25517	COG3428	S	Uncharacterized membrane protein YdbT, contains bPH2 (bacterial pleckstrin homology) domain
25538	COG1309	K	DNA-binding transcriptional regulator, AcrR family
25540	COG1874	G	Beta-galactosidase GanA
25541	COG1309	K	DNA-binding transcriptional regulator, AcrR family
25542	COG2211	G	Na+/melibiose symporter or related transporter
25543	COG5520	M	O-Glycosyl hydrolase
25544	COG1309	K	DNA-binding transcriptional regulator, AcrR family
25547	COG2730	G	Aryl-phospho-beta-D-glucosidase BglC, GH1 family
25548	COG3507	G	Beta-xylosidase
25562	COG1132	V	ABC-type multidrug transport system, ATPase and permease component
25593	COG0101	J	tRNA U38,U39,U40 pseudouridine synthase TruA
25594	COG0203	J	Ribosomal protein L17
25598	COG0361	J	Translation initiation factor IF-1
25628	COG0346	Q	Catechol 2,3-dioxygenase or other lactoylglutathione lyase family enzyme
25656	COG0812	M	UDP-N-acetylenolpyruvoylglucosamine reductase
25663	COG2331	R	Predicted nucleic acid-binding protein, contains Zn-ribbon domain
25675	COG0573	P	ABC-type phosphate transport system, permease component
25676	COG0226	P	ABC-type phosphate transport system, periplasmic component
25680	COG0722	E	3-deoxy-D-arabino-heptulosonate 7-phosphate (DAHP) synthase
25692	COG0184	J	Ribosomal protein S15P/S13E

Table continued

ortholog cluster ID	COG ID	COG category	COG description
25694	COG0366	G	Glycosidase
25707	COG0250	K	Transcription antitermination factor NusG
25708	COG0690	U	Preprotein translocase subunit SecE
25720	COG1136	M	ABC-type lipoprotein export system, ATPase component
25722	COG0681	U	Signal peptidase I
25738	COG1193	L	dsDNA-specific endonuclease/ATPase MutS2
25756	COG0228	J	Ribosomal protein S16
25765	COG2186	K	DNA-binding transcriptional regulator, FadR family
25766	COG3265	G	Gluconate kinase
25767	COG2610	GR	H+/gluconate symporter or related permease
25768	COG1023	G	6-phosphogluconate dehydrogenase (decarboxylating)
25784	COG0065	E	Homoaconitase/3-isopropylmalate dehydratase large subunit
25796	COG1080	G	Phosphoenolpyruvate-protein kinase (PTS system EI component in bacteria)
25801	COG1609	K	DNA-binding transcriptional regulator, LacI/PurR family
25816	COG2217	P	Cation transport ATPase
25816	COG3350	S	Uncharacterized conserved protein, YHS domain
25816	COG4633	R	Plastocyanin domain containing protein
25848	COG0095	H	Lipoate-protein ligase A
25849	COG2314	S	Uncharacterized membrane protein YozV, TM2 domain
25863	COG3304	S	Uncharacterized membrane protein YccF, DUF307 family
25927	COG1086	MO	NDP-sugar epimerase, includes UDP-GlcNAc-inverting 4,6-dehydratase FlaA1 and capsular polysaccharide biosynthesis protein EpsC
25927	COG2148	M	Sugar transferase involved in LPS biosynthesis (colanic, teichoic acid)
25953	COG0613	R	Predicted metal-dependent phosphoesterase TrpH, contains PHP domain
25957	COG5282	S	Uncharacterized conserved protein, DUF2342 family
25996	COG3757	M	Lyzozyme M1 (1,4-beta-N-acetylmuramidase), GH25 family
25998	COG1215	N	Glycosyltransferase, catalytic subunit of cellulose synthase and poly-beta-1,6-N-acetylglucosamine synthase
26000	COG3867	G	Arabinogalactan endo-1,4-beta-galactosidase
26000	COG5183	O	E3 ubiquitin-protein ligase DOA10
26000	COG5263	G	Glucan-binding domain (YG repeat)
26001	COG3594	G	Fucose 4-O-acetylase or related acetyltransferase
26002	COG1216	G	Glycosyltransferase, GT2 family
26003	COG3475	I	Phosphorylcholine metabolism protein LicD
26004	COG1211	I	2-C-methyl-D-erythritol 4-phosphate cytidylyltransferase
26005	COG0451	M	Nucleoside-diphosphate-sugar epimerase
26010	COG4990	S	Uncharacterized protein YvpB
26010	COG5263	G	Glucan-binding domain (YG repeat)
26011	COG1216	G	Glycosyltransferase, GT2 family
26013	COG1260	I	Myo-inositol-1-phosphate synthase
26065	COG2211	G	Na+/melibiose symporter or related transporter
26070	COG0671	I	Membrane-associated phospholipid phosphatase
26072	COG1472	G	Periplasmic beta-glucosidase and related glycosidases
26076	COG4932	S	Uncharacterized surface anchored protein
26082	COG3279	KT	DNA-binding response regulator, LytR/AlgR family
26097	COG0768	DM	Cell division protein FtsI/penicillin-binding protein 2
26097	COG1472	G	Periplasmic beta-glucosidase and related glycosidases
26109	COG0494	V	8-oxo-dGTP pyrophosphatase MutT and related house-cleaning NTP pyrophosphohydrolases, NUDIX family
26119	COG1846	K	DNA-binding transcriptional regulator, MarR family
26119	COG2865	K	Predicted transcriptional regulator, contains HTH domain
26128	COG0230	J	Ribosomal protein L34
26157	COG0705	O	Membrane associated serine protease, rhomboid family
26175	COG2197	TK	DNA-binding response regulator, NarL/FixJ family, contains REC and HTH domains
26178	COG0697	GER	Permease of the drug/metabolite transporter (DMT) superfamily
26185	COG0252	JU	L-asparaginase/archaeal Glu-tRNAGln amidotransferase subunit D
26190	COG1511	S	Uncharacterized membrane protein YhgE, phage infection protein (PIP) family
26208	COG2755	E	Lysophospholipase L1 or related esterase
26212	COG0577	V	ABC-type antimicrobial peptide transport system, permease component
26212	COG1511	S	Uncharacterized membrane protein YhgE, phage infection protein (PIP) family
26213	COG0475	P	Kef-type K+ transport system, membrane component KefB
26221	COG0476	H	Molybdopterin or thiamine biosynthesis adenylyltransferase
26257	COG3937	QT	Polyhydroxyalkanoate synthesis regulator phasin
26266	COG1609	K	DNA-binding transcriptional regulator, LacI/PurR family
26271	COG3534	G	Alpha-L-arabinofuranosidase
26277	COG0452	H	Phosphopantothenoylcysteine synthetase/decarboxylase
26284	COG0626	E	Cystathionine beta-lyase/cystathionine gamma-synthase
26285	COG0514	L	Superfamily II DNA helicase RecQ
26289	COG0787	M	Alanine racemase
26293	COG0480	J	Translation elongation factor EF-G, a GTPase
26293	COG3688	R	Predicted RNA-binding protein containing a PIN domain
26294	COG0456	J	Ribosomal protein S18 acetylase RimI and related acetyltransferases
26303	COG1609	K	DNA-binding transcriptional regulator, LacI/PurR family
26311	COG1893	H	Ketopantoate reductase
26312	COG1464	P	ABC-type metal ion transport system, periplasmic component/surface antigen
26313	COG1135	E	ABC-type methionine transport system, ATPase component
26314	COG2011	E	ABC-type methionine transport system, permease component
26315	COG0436	E	Aspartate/methionine/tyrosine aminotransferase
26346	COG0152	F	Phosphoribosylaminoimidazole-succinocarboxamide synthase

Table continued

ortholog cluster ID	COG ID	COG category	COG description
26352	COG0048	J	Ribosomal protein S12
26356	COG0050	J	Translation elongation factor EF-Tu, a GTPase
26357	COG0231	J	Translation elongation factor P (EF-P)/translation initiation factor 5A (eIF-5A)
26358	COG0781	K	Transcription termination factor NusB
26381	COG1075	I	Triacylglycerol esterase/lipase EstA, alpha/beta hydrolase fold
26393	COG4842	S	Uncharacterized conserved protein YukE
26397	COG1278	K	Cold shock protein, CspA family
26405	COG0765	E	ABC-type amino acid transport system, permease component
26405	COG0834	ET	ABC-type amino acid transport/signal transduction system, periplasmic component/domain
26406	COG1126	E	ABC-type polar amino acid transport system, ATPase component
26417	COG4166	E	ABC-type oligopeptide transport system, periplasmic component
26425	COG0758	L	Predicted Rossmann fold nucleotide-binding protein DprA/Smf involved in DNA uptake
26429	COG2240	H	Pyridoxal/pyridoxine/pyridoxamine kinase
26433	COG4720	S	Uncharacterized membrane protein
26434	COG4172	Q	ABC-type microcin C transport system, duplicated ATPase component YejF
26435	COG0619	H	Energy-coupling factor transporter transmembrane protein EcfT
26436	COG1522	K	DNA-binding transcriptional regulator, Lrp family
26450	COG1757	C	Na+/H+ antiporter NhaC
26452	COG2768	S	Uncharacterized Fe-S center protein
26453	COG0572	F	Uridine kinase
26459	COG1609	K	DNA-binding transcriptional regulator, LacI/PurR family
26460	COG3633	E	Na+/serine symporter

Supplementary Table 8: The list of level 6 ortholog clusters with the COG annotation.

ortholog cluster ID	COG ID	COG category	COG description
24672	COG1473	R	Metal-dependent amidase/aminoacylase/carboxypeptidase
25072	COG1835	M	Peptidoglycan/LPS O-acetylase OafA/YrhL, contains acyltransferase and SGNH-hydrolase domains
25447	COG0242	J	Peptide deformylase
25937	COG1609	K	DNA-binding transcriptional regulator, LacI/PurR family
26127	COG0594	J	RNase P protein component

Supplementary Table 9: Ratio (%) of fecal microbial community by 16S rRNA gene analysis.

	gorilla15 S82	gorilla16 S83	gorilla26 S72	gorilla6 S73	gorilla8 S75	average
Lachnospiracea incertae sedis	29.406	14.627	23.971	24.047	17.945	21.018
Olsenella	3.857	5.405	15.034	8.547	8.255	8.956
Pseudobutyrivibrio	0.006	19.165	0.001	4.065	8.776	7.199
Prevotella	6.833	8.796	3.407	6.778	7.737	6.612
Clostridium IV	7.017	2.487	5.697	5.164	10.18	5.912
Butyrivibrio	6.496	2.255	4.61	4.969	3.034	3.973
Anaerovorax	4.509	2.43	6.318	2.722	3.056	3.787
Oscillibacter	3.915	1.594	2.512	2.266	3.717	2.63
Dialister	2.119	1.807	3.849	2.718	2.217	2.628
Ruminococcus	0.392	5.72	0.694	2.164	2.268	2.485
Rikenella	6.596	2.403	1.113	2.415	1.998	2.374
Clostridium XlVa	1.68	3.517	0.984	2.112	1.652	2.019
Treponema	1.042	1.87	2.585	2.161	1.646	1.989
Subdivision5 genera incertae sedis	1.086	0.54	3.767	1.327	1.157	1.697
Slackia	2.309	0.775	0.619	1.786	2.45	1.448
Bulleidia	0.454	1.228	2.568	0.693	1.539	1.446
Vampirovibrio	0.02	4.209	0.037	1.603	0.083	1.35
Erysipelotrichaceae incertae sedis	0.357	1.024	2.347	0.893	1.256	1.317
Faecalibacterium	0.17	2.08	1.575	0.62	1.311	1.305
Dorea	0.38	2.045	0.467	1.54	1.405	1.255
Sporobacter	2.297	0.757	0.546	0.935	2.418	1.235
Butyricicoccus	0.951	1.088	1.044	1.852	0.564	1.116
Acetivibrio	1.188	1.252	0.758	1.301	0.793	1.035
Blautia	0.556	0.436	2.739	0.339	0.584	1.034
Coprococcus	1.206	0.722	0.601	1.45	0.894	0.927
Roseburia	0.729	0.862	1.13	0.967	0.579	0.879
Eubacterium	0.653	1.46	0.178	0.828	0.812	0.793
Flavonifractor	1.51	0.369	1.055	0.726	0.563	0.761
Sarcina	0.702	0.091	0.001	1.351	1.375	0.666
Anaerostipes	0.155	0.807	0.135	1.127	0.69	0.623
Pseudoflavonifractor	1.188	0.435	0.19	0.261	1.105	0.547
Coriobacterium	0.65	0.928	0.446	0.276	0.436	0.538
Succinivibrio	1.018	0.271	0.32	0.698	0.713	0.536
Robinsoniella	0.094	0.386	0.288	1.22	0.349	0.505
Enterorhabdus	0.416	0.438	0.294	0.428	0.652	0.443
Eggerthella	0.433	0.227	0.527	0.769	0.171	0.424
Oribacterium	0.404	0.381	0.512	0.486	0.255	0.411
Anaerovibrio	0.012	0.056	1.373	0.089	0.1	0.398
Anaerotruncus	0.556	0.289	0.721	0.119	0.253	0.377
Phascolarctobacterium	0.448	0.487	0.307	0.354	0.324	0.375
Clostridium XVIII	0	0.846	0.007	0.802	0.001	0.37
Lactobacillus	0.023	0.32	0.021	1.139	0.01	0.327
Moryella	0.48	0.193	0.315	0.153	0.546	0.316
Xylanibacter	0.029	0.182	0.588	0.188	0.213	0.278
Collinsella	0.158	0.101	0.456	0.168	0.318	0.256
Bifidobacterium	0.173	0.108	0.496	0.173	0.192	0.243
Victivallis	0.246	0.134	0.595	0.085	0.1	0.242
Gordonibacter	0.664	0.168	0.158	0.203	0.249	0.235
Spirochaeta	0.375	0.209	0.118	0.231	0.339	0.234
Acidaminobacter	0.503	0.167	0.103	0.25	0.157	0.197
Paludibacter	0.029	0.068	0.026	0.62	0.106	0.18
Anaerorhabdus	0.424	0.123	0.159	0.11	0.179	0.168
Syntrophococcus	0.5	0.11	0.08	0.168	0.168	0.163
Hallella	0.208	0.155	0.124	0.195	0.14	0.158
Ethanoligenens	0.012	0.083	0.107	0.116	0.356	0.148
TM7 genera incertae sedis	0.319	0.149	0.003	0.172	0.208	0.145
Mogibacterium	0.369	0.088	0.064	0.156	0.143	0.134
Streptophyta	0.067	0.034	0.299	0.025	0.079	0.111
Barnesiella	0.018	0.151	0.093	0.11	0.11	0.107
Anaerofustis	0.102	0.088	0.123	0.059	0.135	0.102
Subdoligranulum	0	0.149	0	0.066	0.135	0.077
Fibrobacter	0.196	0.025	0.011	0.004	0.105	0.049
Clostridium XlVb	0.149	0.025	0.026	0.037	0.069	0.049
Oxalobacter	0.386	0.024	0	0.001	0.037	0.049
Methanosphaera	0.067	0.014	0.058	0.047	0.059	0.046
Streptococcus	0.026	0.058	0.014	0.092	0.026	0.044
Atopobium	0.032	0.033	0.028	0.045	0.079	0.044
Selenomonas	0.003	0.005	0.142	0.006	0.006	0.04
Peptococcus	0.12	0.023	0.037	0.021	0.046	0.04
Parasutterella	0.015	0.028	0.039	0.03	0.037	0.032
Paraprevotella	0.047	0.042	0.024	0.026	0.022	0.03
Campylobacter	0.047	0.023	0.035	0.011	0.014	0.023
Denitrobacterium	0.009	0.06	0	0.022	0.015	0.023
Solobacterium	0.023	0.005	0.06	0.009	0.004	0.021
Gemmiger	0	0.027	0	0.053	0.009	0.02
Herminiimonas	0	0.029	0.002	0.001	0.052	0.019
Escherichia/Shigella	0.006	0.011	0.017	0.04	0.002	0.016
Anaeroplasma	0.003	0.01	0.009	0.011	0.028	0.013
Gracilibacter	0.009	0.012	0.003	0.015	0.023	0.013
Elusimicrobium	0.117	0	0	0	0	0.011
Others	0.237	0.205	0.239	0.172	0.173	0.203

Supplementary Table 10: Classification according to the Carbohydrate Active Enzymes (CAZy) system.

	B.moukalabense GG01T PRJDB7909	*B.moukalabense* GG01T AZMV00000000	*B.moukalabense* GB01	*B.moukalabense* GB03	*B.moukalabense* GB04	*B.moukalabense* GB62	*B.moukalabense* GB63	*B.moukalabense* GB65	*B.moukalabense* CD14	*B.moukalabense* CD16	*B.moukalabense* CD33	*B.moukalabense* EB43	*B.moukalabense* EB44	*B. dentium* Bd1	*B. dentium* ATCC27678	*B. dentium* ATCC27679	*B. dentium* JCVIHMP022	*B. catenulatum* DSM16992	*B. pseudo-catenulatum* DSM20438	*B. longum* DJO10A
CBM6	0	0	0	0	0	0	0	0	0	0	0	0	0	0	0	0	0	0	0	1
CBM22	0	0	0	0	0	0	0	0	0	0	0	0	0	0	0	0	0	0	0	1
CBM23	2	2	2	2	2	2	2	2	2	2	2	2	2	2	2	2	2	0	0	0
CBM26	0	0	0	0	0	0	0	0	0	0	0	0	0	0	0	0	0	0	2	0
CBM32	0	0	0	0	0	0	0	0	0	0	0	0	0	0	0	0	0	0	0	1
CBM35	0	0	0	0	0	0	0	0	0	0	0	0	0	0	0	0	0	1	0	0
CBM41	1	1	1	1	1	1	1	1	1	1	1	1	1	1	1	1	1	0	1	0
CBM46	0	0	0	0	0	0	0	0	0	1	0	0	1	1	1	1	1	0	0	0
CBM48	1	1	1	1	1	1	1	1	1	1	1	1	1	1	1	1	1	1	1	1
CBM61	0	0	0	0	0	0	0	0	0	0	0	0	0	0	0	0	0	0	0	1
CBM67	2	2	2	2	2	2	2	2	2	2	2	2	2	1	1	1	1	0	0	0
CBM74	0	0	0	0	0	0	0	0	0	0	0	0	0	0	0	0	0	0	1	0
CE1	2	2	2	2	2	1	1	1	1	2	1	1	2	1	1	1	1	1	0	1
CE2	1	1	1	1	1	1	1	1	1	1	1	1	1	1	1	1	1	0	0	0
CE4	1	1	1	1	1	1	1	1	1	1	1	1	1	1	1	1	1	0	0	0
CE9	0	0	0	0	0	1	1	1	1	0	0	1	0	1	1	2	2	1	1	1
CE10	2	2	2	2	2	2	2	3	3	2	2	3	2	3	3	5	5	2	4	5
GH1	1	1	1	1	1	1	1	1	1	1	1	1	1	2	2	2	2	0	0	0
GH2	5	5	5	5	5	5	5	5	5	5	5	5	5	6	6	6	6	3	4	1
GH3	15	15	15	15	15	13	13	15	14	13	14	14	13	11	12	12	12	6	5	3
GH5	3	3	3	3	3	3	3	3	3	4	3	3	4	4	4	4	4	2	2	1
GH8	0	0	0	0	0	0	0	0	0	0	0	0	0	0	0	0	0	1	2	0
GH13	14	14	14	14	14	14	14	14	14	14	14	14	14	13	13	14	14	7	15	8
GH20	0	0	0	0	0	0	0	0	0	0	0	0	0	0	0	0	0	0	1	1
GH23	1	1	1	1	1	0	0	1	0	1	1	0	1	0	0	0	0	0	1	0
GH25	2	2	2	2	2	2	2	1	2	1	1	2	1	2	2	2	2	1	2	1
GH26	2	2	2	2	2	2	2	2	2	2	2	2	2	2	2	2	2	0	0	0
GH27	1	1	1	1	1	1	1	1	1	1	1	1	1	0	0	1	1	0	0	1
GH29	1	1	1	1	1	1	1	1	1	1	1	1	1	1	1	1	1	0	0	0
GH30	1	1	1	1	1	1	1	1	1	1	1	1	1	1	1	1	1	1	1	1
GH31	3	3	3	3	3	4	4	2	4	3	3	4	3	4	4	4	4	1	2	3
GH32	1	1	1	1	1	1	1	1	1	1	1	1	1	2	2	2	2	2	1	1
GH33	0	0	0	0	0	0	0	0	0	0	0	0	0	0	0	1	1	0	0	0
GH36	4	4	4	4	4	4	4	4	4	4	4	4	4	5	5	5	5	3	2	1
GH38	0	0	0	0	0	0	0	0	0	0	0	0	0	0	0	0	0	1	0	3
GH42	4	4	4	4	4	4	4	4	4	4	4	4	4	4	4	4	4	3	3	2
GH43	9	9	9	9	9	8	8	8	9	6	6	9	6	10	10	10	10	7	13	10
GH51	2	2	2	2	2	2	2	2	2	2	2	2	2	2	2	1	1	3	3	5
GH53	2	2	2	2	2	2	2	2	2	2	2	2	2	2	2	2	2	0	0	1
GH77	2	2	2	2	2	2	2	2	2	2	2	2	2	2	2	2	2	2	2	2
GH78	2	2	2	2	2	2	2	2	2	2	2	2	2	1	1	1	1	0	0	0
GH85	0	0	0	0	0	0	0	0	0	0	0	0	0	0	0	0	0	0	0	1
GH94	1	1	1	1	1	1	1	1	1	1	1	1	1	1	1	1	1	0	0	0
GH95	0	0	0	0	0	0	0	0	0	0	0	0	0	0	0	0	0	0	1	0
GH101	0	0	0	0	0	0	0	0	0	0	0	0	0	0	0	0	0	0	0	1
GH105	0	0	0	0	0	0	0	1	1	0	0	1	0	0	0	0	0	0	0	0
GH112	0	0	0	0	0	0	0	0	0	0	0	0	0	0	0	0	0	0	0	1
GH120	0	0	0	0	0	0	0	0	0	0	0	0	0	0	0	0	0	1	2	1
GH121	0	0	0	0	0	0	0	0	1	0	0	1	0	1	1	0	0	1	1	0
GH125	1	1	1	1	1	1	1	1	1	1	1	1	1	1	1	1	1	0	0	1
GH127	1	1	1	1	1	1	1	1	2	1	1	2	1	2	2	1	1	3	1	1
GH129	0	0	0	0	0	0	0	0	0	0	0	0	0	0	0	0	0	0	0	1
GT2	9	9	10	10	9	6	6	10	6	7	6	6	7	15	16	7	8	7	6	5
GT4	8	7	8	8	8	3	3	7	2	5	3	2	5	8	8	5	3	7	6	2
GT5	1	1	1	1	1	1	1	1	1	1	1	1	1	1	1	1	1	1	1	1
GT14	0	0	0	0	0	0	0	0	0	0	0	0	0	1	1	1	1	0	0	0
GT28	1	1	1	1	1	1	1	1	1	1	1	1	1	1	1	1	1	1	1	1
GT32	0	0	0	0	0	0	0	0	0	0	0	0	0	0	0	0	0	0	1	0
GT35	1	1	1	1	1	1	1	1	1	1	1	1	1	1	1	1	1	1	1	1
GT39	1	1	1	1	1	1	1	1	1	1	1	1	1	1	1	1	1	0	0	0
GT51	2	2	2	2	2	2	2	2	2	2	2	2	2	2	2	2	2	2	2	2
